# How unequal is unequal? Turn-taking simulation for benchmarking participation and opportunity inequality in small-group collaboration

**DOI:** 10.1371/journal.pone.0361205

**Published:** 2026-10-23

**Authors:** Liru Hu, Jiajun Wu, Feng Yu, Deliang Wang, Song An, Ying Zhang

**Affiliations:** 1 College of Education, University of Texas at El Paso, El Paso, Texas, United States of America; 2 Faculty of Education, University of Hong Kong, Hong Kong, China; 3 Department of Mathematical Sciences, University of Texas at El Paso, El Paso, Texas, United States of America; 4 Department of Mathematics and Information Technology, The Education University of Hong Kong, Hong Kong, China; Universitas Prima Indonesia, INDONESIA

## Abstract

Participation equity is essential for ensuring that students gain cognitive and social benefits from collaborative learning, yet there is a lack of quantitative benchmarks for interpreting participation and opportunity distributions in small-group collaboration. This study focuses on two turn-based analytical dimensions of participation equity: participation equality, measured by students’ shares of speaking turns, and opportunity equality, measured by students’ shares of addressed opportunities. Using the standard deviation of these rates, the study establishes preliminary, context-specific simulation-based reference values for identifying departures from an idealized triadic turn-taking baseline. The empirical dataset included 117 fourth-grade primary school students from two classes in mainland China, who worked in heterogeneous triads on mathematical and reading tasks. Using agent-based modeling and Monte Carlo simulation, the study estimated local turn-taking parameters from empirical data, generated reference distributions under an ideal-equity baseline, and conducted sensitivity analyses to examine model stability. Results support the internal stability of the model and provide turn-count-specific reference values for interpreting participation and opportunity inequality. The findings offer preliminary reference values for interpreting participation and opportunity distributions in collaborative learning and highlight the importance of turn count when evaluating structural patterns of participation and opportunity in triadic classroom dialogue. These reference values describe structural participation patterns rather than collaboration quality and require recalibration and independent validation before application in other educational settings.

## Introduction

In a recent peer talk intervention for primary school fourth graders [1], triads were provided with a “windmill chart” visualizing the number of turns spoken by each member (see Fig 1). When asked if they had participated equally, the students consistently replied, “No!” Their reasoning was simple: “Because the three leaves of the windmill are not the same!”

**Fig 1:**
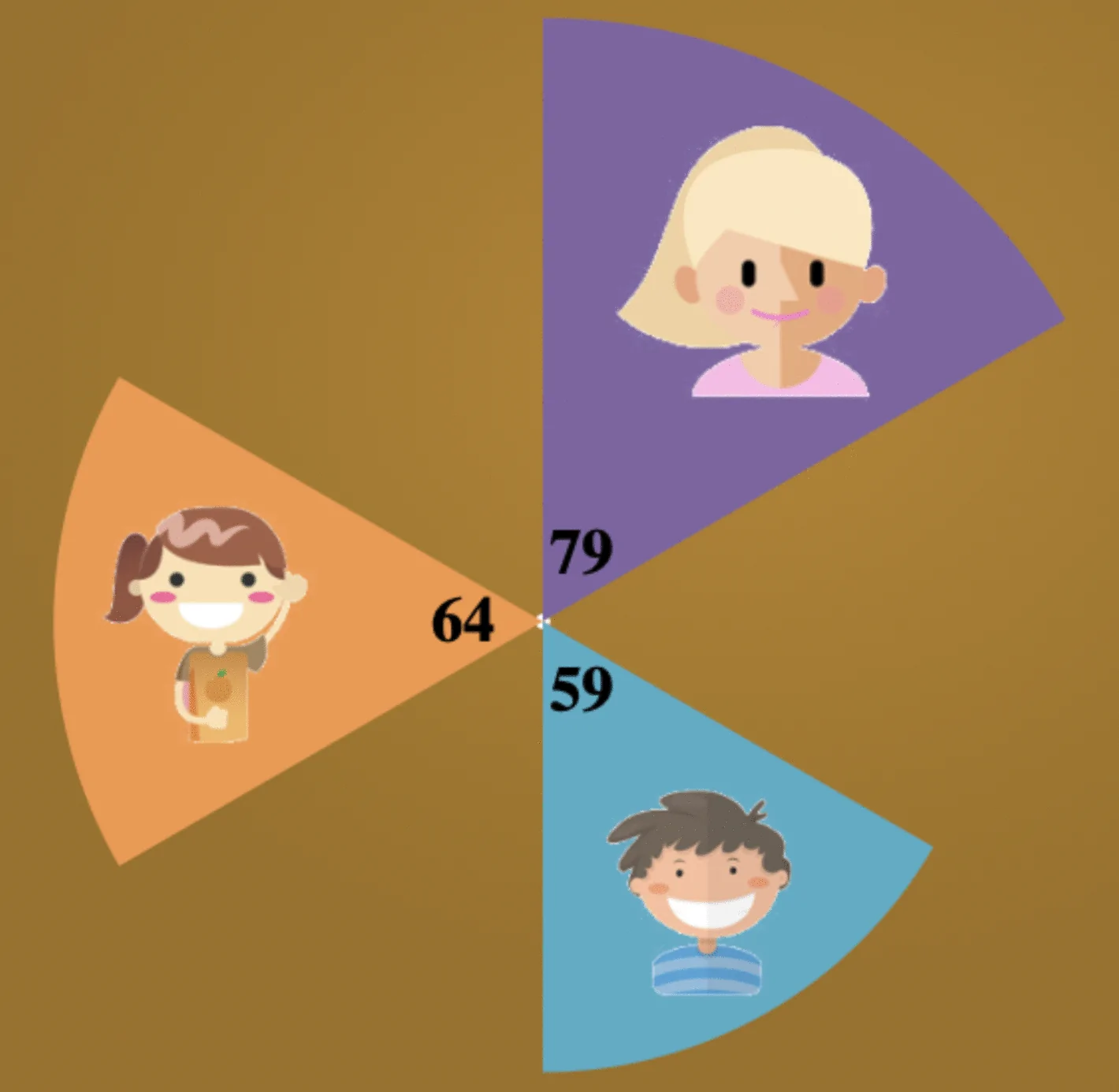
Example of a participation windmill chart. *Note.* Each leaf represents one group member. The numerical value indicates the number of spoken turns produced by that member, and the area of each leaf is proportional to the turn count.

This example illustrates a challenge in interpreting visual participation feedback: numerical differences in spoken turns are visible, but there is no guidance on whether such differences fall within a reasonable range of natural conversational variation. For these young learners, any numerical disparity in turn-taking equated to unequal participation. In contrast, teachers generally recognize that balanced participation does not require perfect numerical identity. However, they often lack a clear, evidence-based standard to identify what degree of variation is potentially acceptable. A statistical reference can help interpret variation under specified turn-taking assumptions, while judgments about educationally meaningful inequality also require contextual and qualitative evidence. This observation raises a fundamental question: What quantitative reference values can help distinguish natural variation in participation from potentially meaningful imbalance, and how can we establish these metrics to better guide collaborative learning?

## Literature review

### Significance of equity in collaboration

Equity in collaboration is crucial for unlocking the full potential of learning through specific forms of dialogue, a dialogic approach widely recognized for its effectiveness in enhancing students’ learning, comprehension, and skill development [2–4]. However, adopting a dialogic educational approach often encounters significant challenges, particularly when issues of social dominance and isolation arise within group interactions [5–8]. Inequitable participation during collaborative activities can have detrimental effects on both group-level and individual-level outcomes, impacting cognitive and affective dimensions of learning.

At the group level, balanced participation is associated with several features of productive collaboration. More evenly distributed participation has been linked to greater satisfaction in online collaboration [9], while temporal patterns of equal and active participation have been positively related to team learning gains [10]. Psychological safety and interpersonal trust can further support students’ willingness to contribute to group work [11]. Equality in conversational turn-taking has also been associated with higher collective intelligence across diverse group tasks [12]. In computer-supported collaborative learning, productive collaboration likewise depends on groups’ ability to monitor and regulate collective knowledge-building processes [13].

At the individual level, however, the relationship between vocal participation and learning is less straightforward. Providing detailed explanations and engaging with peers’ mathematical ideas have been associated with achievement [2]. Vocal contribution alone need not predict learning: O’Connor et al. [7], for example, found no relationship between how much an individual student spoke and learning outcomes in classrooms where a culture of active participation had been established. Unequal vocal participation should therefore not automatically be interpreted as unequal learning. Nevertheless, participation imbalances may still signal problematic collaborative experiences. In online collaborative learning, perceived asymmetry among group members has been identified as an important source of student frustration [14], while lower satisfaction with online learning has been associated with greater disengagement [15]. Thus, participation inequity warrants attention as a feature of the collaborative process even when unequal speaking rates do not necessarily imply unequal individual learning.

### Quantitative representation of participation equity

Recognizing the importance of participation equity, efforts to cultivate equitable peer interaction have been undertaken through various means. One such effort is to provide group members with visual representation of their participation distribution in social group awareness tools [16,17], collaboration analytics tools [18] and teacher orchestration tools [19]. These tools utilize different types of data and visualization approaches to achieve their objectives to promote awareness of participation equity and encourage balanced engagement during collaborative activities [6,20].

For instance, Reflect is an interactive table designed to provide real-time shared visualization of group participation levels. This tool uses multicolor Light-Emitting Diodes (LEDs) to represent each participant’s accumulated speaking time [21]. Reflect improved users’ awareness of speaking-time shares; its effect on participation balance was statistically significant among participants who considered balanced participation important.

EQUIP is an observation tool aimed at monitoring and visualizing the verbal participation of diverse demographic groups in classroom settings [22]. It employs bar charts to display the distribution of contributions across different demographic categories, such as gender or ethnicity. EQUIP demonstrates how disaggregated participation data can reveal demographic patterns for further interpretation.

Similar tools also include Second Messenger which uses visualizations to represent patterns of participation within groups, enabling members to recognize dominance or isolation in conversations [23] and Conversation Clock which visualizes conversational dynamics through a clock-like interface, showing when and how frequently each participant contributes [24]. Most of these initiatives focus on promoting balanced participation among individuals or demographic groups by visually displaying the distribution of participation data among group members [17,25,26]. Visual analytics systems also support inspection of online forum activity, as in iForum [27], while teacher orchestration tools aim to help teachers monitor collaboration and decide when support may be needed [19].

While these tools provide graphical representations of interaction patterns, they largely leave the interpretation of this information to users, whether students or teachers [16,19,20]. This reliance on open scaffolding highlights the inherent complexity of determining fairness solely based on the quantitative distribution of participation rates. For example, a balanced distribution of participation rates may not necessarily equate to fairness, as powerful or disrespectful language such as issuing commands or displaying rudeness can undermine equitable group dynamics [1,8]. Moreover, participation rates are influenced by factors such as academic competence, task demands, and personality traits, which can cause these rates to fluctuate dynamically over time. Thus, achieving equitable participation often requires time and sustained effort.

Treating equal participation as a definitive learning objective is also problematic. Silence during collaboration does not always signify disengagement, as it may reflect deeper cognitive processing or active listening [7,28]. However, not all silence is productive, and educators must carefully evaluate whether silent behaviors contribute to learning or indicate a need for intervention. Therefore, unequal vocal participation should still warrant attention, even when productive silence is present. The potential value of group-awareness feedback depends in part on how users interpret and act on it [25,29]. Younger students, in particular, may struggle to interpret participation-equity visualizations [1] while adults’ interpretations of such visualizations [30] and teachers’ dashboard-use practices [31] can also vary.

Teacher-facing notifications have been used to draw attention to groups’ progress, misconceptions, and critical moments [32,33]. However, determining when and how to implement such scaffolds remains a challenge. Alerts can sometimes impair teachers’ observations of collaborating groups [34], making their timing and relevance important design considerations. These challenges emphasize the importance of developing structured guidance to accompany visual representations, ensuring that users can effectively interpret participation data and take appropriate actions to address participation inequity.

### Absence of benchmarks for quantitative metrics

Researchers have developed various metrics to evaluate participation equity, typically represented as the degree of variability in the distribution of participation units, such as turns, comments, lines of text, or word counts [35,36]. These metrics include the Gini coefficient [9,37], adjusted versions of the Gini coefficient [36], standard deviation (SD) [38,39], and the coefficient of variation [36,40]. Recent studies further highlight that participation can be operationalized in multiple quantitative ways, making metric selection a consequential methodological decision rather than a merely technical one. For example, Asano et al. [41] evaluated participation balance using turns, words, and speech duration as participation units, and compared SD, absolute deviation, concentration, adjusted Gini, and entropy-based measures. Similarly, Peng et al. [10] examined temporal participation equality using a normalized coefficient-of-variation measure based on pairwise turn-taking counts within adaptively identified discussion segments.

Moreover, these metrics are not directly interchangeable. The Gini coefficient has been widely used to summarize the concentration of participation across group members [9,37]. However, because its maximum attainable value depends on group size, adjusted Gini measures have been proposed to improve comparability across groups of different sizes [36,42]. The SD and coefficient of variation, on the contrary, capture the dispersion in individual participation rates and are relatively intuitive for visual learning analytics because they directly reflect how far members deviate from equal participation. Entropy-based measures emphasize the diversity or uniformity of participation distributions.

The central challenge, therefore, lies not in the availability of quantitative metrics or simply in determining which metric is mathematically preferable. Rather, it remains unclear how a given metric value should be interpreted in relation to discussion length, group structure, and the intended use of the analysis. This interpretive gap becomes especially consequential when learning analytics tools use quantitative metrics to trigger automatic alerts or prompts aimed at regulating participation equity. For example, Strauß and Rummel [9] used a Gini coefficient threshold of 0.50 to trigger adaptive prompts in a group awareness tool. Although this provides an operational cutoff, it does not resolve how such thresholds should be calibrated across different discussion lengths or interaction conditions. Thus, the broader need is for principled, context-sensitive reference values for interpreting participation inequality.

There remains a lack of established reference benchmarks to evaluate the educational meaning of such metrics. Questions such as “what level of SD in individual participation rates signals potential participation inequity?” or “What threshold can meaningfully guide collaboration interventions in learning analytics tools?” remain unanswered. While an ideally equitable context might assume uniform individual participation rates, such perfect equality is often impractical due to various objective factors, including time constraints, group size, and turn-taking mechanisms. In many cases, slight variations in participation may not reflect true inequity, while significant deviations could indicate deeper issues requiring attention. Without benchmarks, educators and researchers lack the tools to distinguish between acceptable variations and potentially problematic imbalances.

Establishing benchmarks is therefore essential for making these metrics actionable. Simulation-based benchmarks can locate an observed imbalance within a specified reference distribution, but cannot by themselves determine whether that imbalance is educationally problematic. Threshold exceedance may serve as a signal for closer examination; determining whether and how to intervene requires additional contextual or educational evidence. They can also inform the design of learning analytics tools, helping students and teachers interpret quantitative data or visual representations more effectively.

### Influencing factors of participation equity

Participation equity is influenced by numerous individual and contextual factors [43], making it even more challenging to provide appropriate prompts about participation equity. First, group size is an important factor shaping participation opportunities and interaction patterns in collaborative learning [42,44]. However, observed relations between group size and participation inequality depend on context and measurement [42]. A comparison of dyads and quads found more peer communication and interaction in dyads [44]. In a different setting, Saqr et al. [45] found that larger online medical-student groups of 7–15 members were less cohesive and showed less efficient communication.

Second, students’ engagement in collaborative activities is closely associated with their individual characteristics. Previous research has highlighted the influence of students’ status within a group on their participation dynamics. Cohen and Lotan [46] identify several indicators of status, including academic status (referring to one’s academic performance ranking within the group), peer status (indicative of attractiveness or popularity among peers resulting from interactions within and outside the school context), and social status (which encompasses distinctions related to social class, gender, race, and ethnic background). Information about students’ status can be collected through demographic surveys that capture parental educational backgrounds, gender, ethnicity, or by soliciting students’ self-reported levels of friendship with fellow group members. Alternatively, academic status can be inferred from recent academic scores.

Furthermore, individual characteristics can be associated with participation patterns, although these associations vary across characteristics and contexts [47]. Students may participate less when they have difficulty articulating their ideas, lack relevant subject knowledge, or fear appearing unintelligent in front of peers or instructors [48]. Personality-related tendencies may also shape group interaction. Anderson and Kilduff [49] found that trait dominance predicted group influence through perceived competence even when actual ability was controlled. Cultural norms and local educational contexts may also influence students’ willingness to speak, ask questions, or challenge others [50,51]. Some of these characteristics, such as self-concept, learning enjoyment, and social anxiety, can be assessed using established psychological scales [47].

Third, different types of tasks can lead to varying levels of participation equity. Ill-structured problems may require students to analyze and organize information, generate solutions, and make decisions, creating a need for appropriate scaffolding [52]. In a direct comparison of problem types, Kapur and Kinzer [53] found that triads solving an ill-structured problem generated more problem-centered interaction than those solving a well-structured problem, but also exhibited greater participation inequity. Task characteristics may also interact with students’ knowledge, competence, and status to shape participation patterns. Multidimensional or open-ended tasks that draw on different competencies can provide multiple opportunities for students to contribute [46,54], while jigsaw activities can distribute responsibility and promote interdependence among group members [55]. Thus, tasks that rely on a narrow set of competencies or prescribed procedures may advantage particular members, whereas tasks offering multiple points of entry may support broader participation.

### Present study

A limitation in the current literature is the predominant focus on promoting equal participation among individuals or demographic groups, often overlooking other essential dimensions of equitable peer interaction. Shah and Lewis [8] define participation equity as “a condition where opportunities to participate—and participation itself—are fairly distributed among all students involved in a learning interaction” (p. 428). Guided by this definition, the present study focuses on two interrelated aspects of participation equity: participation equality and opportunity equality. Participation equality concerns the extent to which individual group members’ actual participation is evenly distributed, whereas opportunity equality concerns the extent to which opportunities to enter or re-enter the conversation are evenly distributed among group members.

In this study, these two aspects are operationalized through turn-based indicators. We measure the level of participation via the number of turns produced by a speaker. One participation opportunity is quantified as a turn directed toward a member, effectively granting them the conversational floor [1]. For instance, in a triadic group consisting of A, B, and C, if Member A addresses Member B, it is assumed that A has granted the conversational floor to B. Therefore, A is credited with participation, while B receives a participation opportunity. Alternatively, if A addresses the entire group rather than specifically targeting B or C, both B and C would each receive an opportunity. As shown in Fig 2, participation and opportunity are therefore treated as separate dimensions: speaking turns capture actual participation, whereas addressed turns capture opportunities made available by peers.

**Fig 2:**
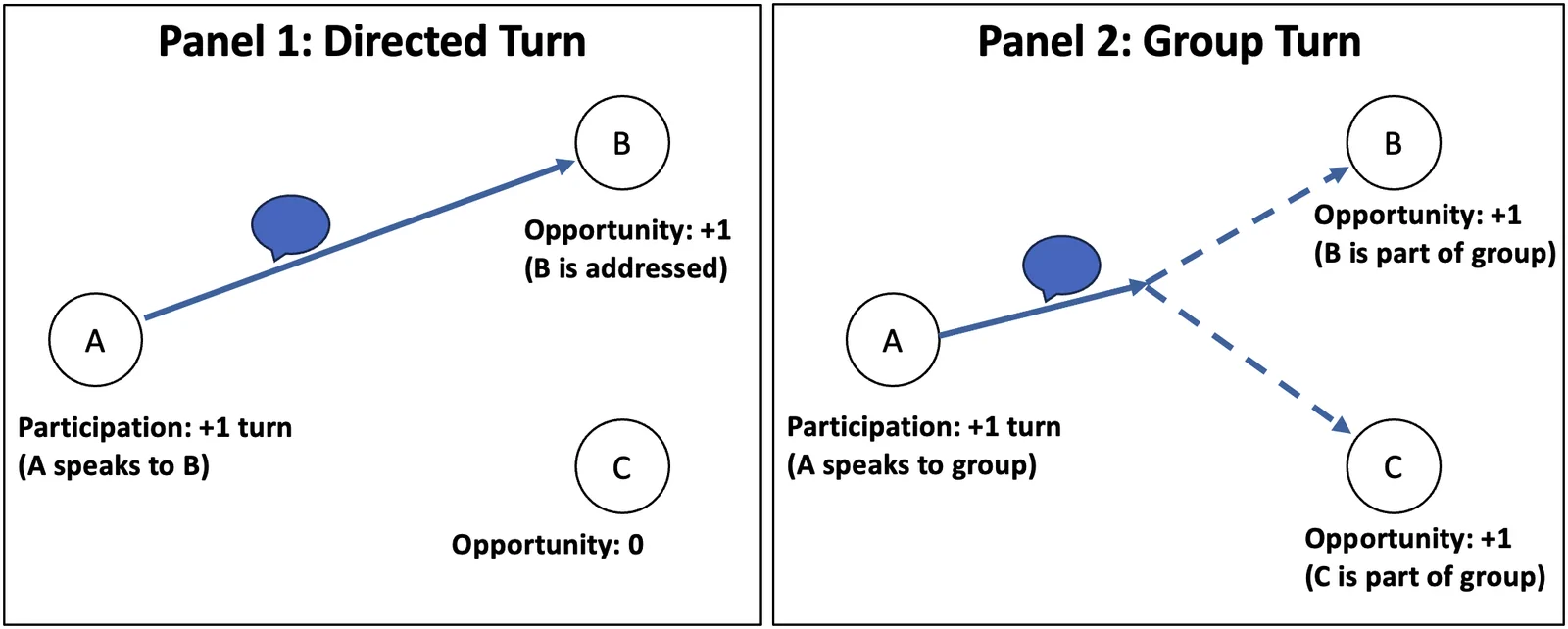
Visualizing participation and opportunity in a triad [1]. *Note.* The speaker receives one participation count, while the addressee receives one opportunity count. When the speaker addresses the whole group, all non-speaking members are counted as receiving an opportunity.

We evaluate these dimensions using the SD of individual shares. Participation inequality is the SD of individual participation rates, defined as each member’s speaking turns divided by total group turns. Similarly, opportunity inequality is the SD of individual opportunity rates, defined as each member’s received opportunities divided by the total addressed opportunities across all three members.

This study seeks to generate preliminary, context-specific reference values for these two metrics that distinguish observed imbalances from variation under the specified turn-taking baseline. To this end, we implement a custom turn-taking simulator in Python using NumPy. Python agent-based modeling and educational simulation provide broader methodological background [56,57].

Empirical classroom data alone cannot provide ground-truth “equal participation” scenarios, because real students inevitably introduce participation differences stemming from competence, personality, anxiety, social status, and other factors. Simulation provides a controlled way to construct idealized reference distributions for the two metrics [58]. By systematically constraining individual- and relationship-specific factors while retaining local turn-transition structures estimated from the empirical data, the model provides an idealized reference distribution for interpreting participation and opportunity variation. In addition, simulation allows precise experimental control over turn-taking patterns, which would be expensive, time-consuming, and often infeasible to implement in small-scale classroom studies. Simulation is also widely used to model dialogic processes and social systems [59,60].

Our analysis focuses specifically on triadic groups, a central and widely used configuration in small-group learning. Triads are the smallest group size in which coalition and third-party dynamics become possible (e.g., two-against-one alignments), creating the minimal structural conditions for dominance and peripherality to emerge. Within this context, the study investigates the following two research questions for triadic groups:

- **Research Question 1:** Beyond which SD of individual participation rates can we flag that a triadic group is experiencing a potential participation inequality issue?
- **Research Question 2:** Beyond which SD of individual opportunity rates can we flag that a triadic group is experiencing a potential opportunity inequality issue?

## Turn-taking modeling

### Individual and interactional sources of participation variation

The simulated turn-taking model distinguishes between two broad sources of participation variation: individual/social factors and local sequential features of dialogue. Individual and social factors refer to relatively stable characteristics or relational conditions that may systematically advantage or disadvantage particular group members in classroom interaction. We classify these factors into two categories: Type I and Type II factors.

Type I factors pertain to elements that impact an individual’s possibility to be the speaker. For instance, one’s self-concept primarily influences their confidence in their academic abilities. Students with lower levels of self-concept may hesitate to express their ideas or comment on others’ contributions due to insecurity about their own ideas and a fear of making errors [47]. Social anxiety is another characteristic that falls within the Type I factors. It is prevalent among young people and may be associated with disruptions in functioning [61]. In small-group problem-based learning contexts, silence can arise from multiple contextual and power-relation factors [48]. Similarly, a hypothetical personality-related difference in students’ propensity to take the speaker role would be modeled as a Type I factor.

Type II factors, on the other hand, represent factors that may influence an individual’s likelihood of being the addressee. Academic status is a potential Type II factor, motivated by the broader concern with status differences in groupwork [46]. For modeling purposes, a hypothetical preference for addressing a peer perceived as academically proficient would alter that peer’s addressee probability. Conversely, the same hypothetical preference would reduce the relative opportunities directed to other members. Friendship is another possible relational influence on whom a student addresses; this is a candidate mechanism rather than an effect estimated in the present study. Psychological safety concerns the perceived interpersonal risks of speaking up [62], but does not by itself establish that friendship determines whom students address in classroom interaction. Relational-event models also represent reciprocity through histories of interaction [63]; applying this concept to classroom addressee selection requires empirical assessment.

### Local sequential parameters in triadic dialogue

Although individual and social factors are central to authentic classroom interaction, human dialogue is not a purely random process even when such factors are minimized. In an ideally equitable group discussion, participants may have equal baseline status and equal opportunity to contribute, but their turns remain organized by local sequential constraints.

Extensive research in conversation analysis and small-group interaction has examined how turns are locally organized in dialogue. Classic turn-taking theory distinguishes between situations in which the current speaker selects the next speaker and situations in which the next speaker self-selects [64]. In group conversation, Gibson’s participation-shift framework further conceptualizes interaction as a sequence of moment-by-moment shifts among participation statuses, including speaker, target/addressee, and unaddressed recipient [65]. More recent formal work on task-group conversation similarly argues that conversational behavior is shaped by both local turn-taking rules and broader status processes, and that turn-level models should account for sending/speaking and receiving/targeting tendencies [47,66]. These perspectives suggest that even an idealized equity model should preserve basic sequential structures of turn allocation and addressivity rather than treating each turn as fully independent.

Accordingly, the present model incorporates three local sequential parameters, as outlined in Table 1. First, *P*_rec_ captures recipient uptake, or the probability that the participant addressed in the previous turn becomes the next speaker. This parameter corresponds to the turn-receiving pattern in which speaker A addresses participant B, and B subsequently takes the conversational floor [47,65,67]. Such recipient-to-speaker transitions are consistent with established principles of turn allocation in conversation, whereby an addressed participant is positioned to take the next turn [64,68,69]. Thus, *P*_rec_ complements Type I factors by representing a local sequential influence on speaker selection.

**Table 1:** Temporal factors influencing turn-taking patterns.

Pattern	Rate	Representation	Interpretation
Turn-receiving	*P*_rec_	A -> (B) - > B - > X	A talks to B, then B talks to X (could be A)
Talk-back	*P*_tb_	A -> (B) - > X -> (A)	A talks to B, then X (could be B) talks back to A
Talk-to-group	*P*_tg_	A -> (Group) - > X	A talks to the group without nominating the next speaker

*Note*. rec = receiving; tb = talk-back; tg = talk-to-group.

Second, *P*_tb_ captures sequential return addressing, defined as the probability that the current speaker addresses the immediately preceding speaker. This parameter represents a local dependency in addressee selection. Conversation-analytic work shows that adjacent turns are sequentially organized, such that a prior turn can make a response relevant in the next position [68]. Participation-shift research likewise shows that transitions in speaker and addressee roles depend on the immediately preceding conversational configuration [65,67]. In triadic discussion, this reciprocal organization may appear when the next speaker directs the following turn back to the previous speaker. Thus, *P*_tb_ complements Type II factors by representing a local sequential influence on addressee selection.

Third, *P*_tg_ captures open-floor addressing, defined as the probability that the current speaker addresses the whole group rather than nominating a specific individual. Multiparty interaction cannot be reduced to a simple speaker-hearer dyad. Participation framework theory distinguishes among speakers, addressed recipients, ratified participants, and other participation statuses [70]. Similarly, work on hearers and speech acts distinguishes utterances directed to particular addressees from utterances made available to all participants [71]. In collaborative group discussion, a speaker may therefore direct a turn to the group as a whole, opening the conversational floor to multiple participants rather than assigning the next opportunity to one member. Thus, *P*_tg_ represents a local addressivity feature that is especially relevant to opportunity equality.

The three parameters are not intended to exhaustively represent the full complexity of collaborative dialogue. Instead, *P*_rec_, *P*_tb_, and *P*_tg_ define a minimal local structure for modeling turn allocation and addressivity in triadic classroom dialogue. They allow the idealized baseline model to remain conversationally realistic without incorporating systematic individual- or status-based asymmetries. When local turn allocation and addressivity operate together with asymmetric and dynamic Type I and Type II factors, higher-order participation structures such as dominance, coalition formation, or role differentiation may emerge. More complex patterns, such as dominance or coalition, can be understood as emerging when Type I/II factors become asymmetric or dynamic over time. In this sense, *P*_rec_, *P*_tb_, and *P*_tg_ provide a stable dialogue-structural baseline, while individual-, relational-, and history-dependent processes can be conceptualized as variations in Type I and Type II probabilities layered onto that baseline.

Furthermore, we distinguish these local sequential parameters from cumulative relational processes. For example, relational-event models define general reciprocity as the tendency for one person to initiate interactions toward another as a function of the volume of prior interactions received from that person [63]. Such cumulative reciprocity represents an evolving, history-dependent relational process rather than an immediate turn-to-turn dependency. We therefore categorize it as a Type II factor rather than as part of the minimal local turn-taking structure used in the present idealized baseline model.

In summary, the turn-taking simulation focuses on three empirically estimable parameters related to local turn-taking tendency. *P*_rec_ influences the selection of speakers by capturing whether an addressed participant takes up the next turn. *P*_tb_ and *P*_tg_ influence the selection of addressees by capturing whether the next turn is oriented back to the previous speaker or opened to the group as a whole. The general decision-making process for turn-taking in the model is presented in Table 2.

**Table 2:** Turn-taking simulation procedure and rules.

Step	Turn	Speaker	Addressee	Criteria of choice
1	1	Speaker_1_		Type I factors
2	1		Addressee_1_	Type II factors
3	2	Speaker_2_		*P*_rec_ and Type I factors
4	2		Addressee_2_	*P*_tg_, *P*_tb_, and Type II factors
⋮	⋮			
2n−1	*n*	Speaker_*n*_		*P*_rec_ and Type I factors
2*n*	*n*		Addressee_*n*_	*P*_tg_, *P*_tb_, and Type II factors

### Idealized equitable baseline simulation

In this study, our focus is on triadic groups, and we aim to simulate ideally equitable group discussions to identify the thresholds for participation equality and opportunity equality. In such ideally equitable discussions, each group member should be treated as equals in collaboration regardless of their individual characteristics or demographics such as ethnicity, academic status, socioeconomic status, personality or friendships with others [8,47,54]. Therefore, we exclusively consider the local sequential parameters (*P*_rec_, *P*_tb_, *P*_tg_) in our turn-taking simulations and do not incorporate personalized factors (Type I and Type II).

This design is a constrained baseline rather than a full model of classroom collaboration. It does not assume that real classroom interaction is free from status, friendship, competence, or personality effects. Instead, it asks a narrower question: when individual-specific advantages are not explicitly represented in the simulator, how much variation in participation and opportunity can arise under a model containing only the specified local turn-taking dependencies? The resulting Monte Carlo simulation provides a reference distribution against which observed classroom groups can be compared. The detailed simulation process for an ideally equitable triadic group discussion unfolds in Algorithm 1. The resulting baseline is most informative when the task offers broadly comparable opportunities for all three members to contribute. Assigned roles, tutoring, extended explanations, or differences in task responsibility can produce expected participation asymmetries, so departure from this baseline does not by itself indicate problematic collaboration. Moreover, excluding individual and relational differences from the simulator does not establish that the empirical transition probabilities used to calibrate the model are free from such influences.

**Algorithm 1 Turn-Taking Simulation Procedure**

1: **Input:** Group members 𝒢, rates *P*_rec_, *P*_tg_, *P*_tb_

2: **Output:** Speaker and addressee for each turn

3: **for** turn t=1,2,...,n **do**

4: **if** *t* = 1 **then**

5:   Select $S_{t}$ uniformly from 𝒢

6:   Select $A_{t}$ uniformly from three targets:

7:    the whole group and the two members other than $S_{t}$

8:  **else**

9:  **if** $A_{t-1}$ is an individual member **then**

10:   With probability *P*_rec_, set $S_{t}=A_{t-1}$

11:   Otherwise, set $S_{t}$ to the member other than $S_{t-1}$ and $A_{t-1}$

12:  **else**

13:   Select $S_{t}$ uniformly from the two members other than $S_{t-1}$

14:  **end if**

15:  Draw a fresh u~Uniform(0,1), independently of speaker selection

16:  **if** *u* < *P*_tg_ **then**

17:   Set $A_{t}=𝒢$

18:  **else if** $u<P_{\text{tg}}+P_{\text{tb}}$ **then**

19:   Set $A_{t}=S_{t-1}$

20:  **else**

21:   Set $A_{t}$ to the member other than $S_{t}$ and $S_{t-1}$

22:  **end if**

23:  **end if**

24: **end for**

## Empirical study

We model the local sequential structure of triadic turn-taking using three probabilities, namely, *P*_rec_, *P*_tb_, and *P*_tg_. These parameters are treated as simplified, empirically estimable approximations of recipient uptake, sequential return, and open-floor addressing in the present classroom dataset. In this section, we estimate these probabilities from our empirical dataset and propose simulation-based reference values for flagging potential participation or opportunity inequality in triadic groups.

The description of our empirical dataset is provided in the Data description subsection, and the estimation of the probabilities is detailed in the Probability estimation subsection. We introduce a new set of measures to quantify inequality in participation and opportunity, and we formulate a criterion based on these measures, in the Participation and opportunity inequality subsection. Using this criterion, we examine the presence of inequalities in our empirical dataset in the Empirical triangulation subsection. The Robustness check using the Gini coefficient subsection examines the robustness of the simulation-based procedure to metric choice by comparing SD-based and Gini-based reference distributions and classifications. Finally, the Stability analysis subsection examines the internal stability of the modeling approach through parameter recovery and one-at-a-time perturbations of the interaction probabilities. These analyses assess whether the simulation reproduces its specified parameters and how sensitive the reference thresholds are to plausible parameter changes; they do not establish educational validity or transferability to other settings.

### Data description

The present study reports a secondary analysis of data collected for the first author’s dissertation project [72]. In the original study, participants were recruited from 15 December 2020 to 5 February 2021. Written informed consent was obtained from all participating teachers and from the parents or legal guardians of student participants prior to data collection, and verbal assent was obtained from student participants in the classroom before participation. The original study was approved by the Human Research Ethics Committee, The University of Hong Kong (Reference No. EA1903032). For the present analysis, the previously collected dataset was accessed for research purposes on 1 September 2024. Although the first author had access to identifiable participant information during the original data collection process, all data used in the present analysis were de-identified before analysis.

The dataset consists of 117 fourth-grade primary school students drawn from two classes located in mainland China. From this dataset, we extracted a final corpus of 245 valid triadic collaborative episodes (*M* = 72 turns per episode, *SD* = 43) [73]. An episode was defined as the complete discussion of a single triad jointly solving a specific task; episodes were excluded from the analysis if audio recordings were incomplete or if the triad configuration was disrupted (e.g., student absence).

To ensure comparable group dynamics, students were organized into heterogeneous triads based on a multi-dimensional assessment of academic and social factors. We first categorized students into three competency levels using a composite score of recent teacher-reported grades in mathematics and Chinese, alongside individual reasoning ability. Triads were then formed by selecting one student from each level. To mitigate status inequities and encourage participation, we incorporated sociometric data in which students nominated preferred partners and peers frequently consulted for mathematics questions. Specifically, group composition prioritized the partner preferences of lower-achieving students when selecting middle- and high-achieving teammates. Final group assignments were adjusted based on teacher input regarding interpersonal relationships to ensure gender balance where feasible and to maximize psychological safety.

The collaborative activity took place across multiple sessions. Students worked sequentially on “process-open” mathematics problems and reading comprehension tasks. The mathematics tasks varied in complexity. Examples included predicting the shape of a tightened knot or creating geometric patterns with triangular tiles. The reading tasks required students to reorder scrambled sentences into coherent narratives. In total, the groups completed five mathematics problems and two reading tasks. There were no strict time limits, although tasks generally lasted about 15 minutes. Before the activity began, teachers established norms for mutual respect and equitable participation. They explicitly informed students that the evaluation would consider the quality of their collaboration in addition to the accuracy of their solutions.

### Probability estimation

We estimate the three parameters in triadic group discussions at the individual level. Specifically, individual *P*_rec_ quantifies how frequently a participant takes the conversational floor after being individually addressed, relative to the number of times that participant is addressed. Individual *P*_tg_ measures how frequently a participant addresses the entire group relative to their total speaking turns. Individual *P*_tb_ quantifies how frequently a participant addresses the immediately preceding speaker relative to their total speaking turns. These individual-level rates can vary across participants and may reflect individual or relational differences in addition to local turn-taking processes. For example, a participant-specific tendency to take the floor more or less often after being addressed would be reflected in variation in individual *P*_rec_.

When estimating the three parameters, we excluded all participant-level rate estimates from a group discussion if any member contributed fewer than 10 speaking turns. The 10-turn cutoff was a pragmatic minimum intended to improve the stability of frequency-based parameter estimates, rather than a criterion for participation quality. With few eligible events, a single event can substantially change an estimated probability; this is particularly relevant to *P*_tg_ and *P*_tb_ when speaking turns are scarce.

Subsequently, we calculated the means of individual rates by averaging them across various types of tasks and different group compositions. Finally, we averaged all rates from all individuals to derive estimates of the parameters. Pooling these rates was intended to reduce dependence on any single task or group composition; it does not imply that individual or relational influences were eliminated from the empirical estimates. The exclusion applied only to parameter estimation; all 245 valid episodes were retained for the empirical assessment. Because the cutoff conditions parameter estimation on discussions with at least minimal participation from all three members, the resulting estimates should be interpreted as calibration values for the baseline model rather than as estimates characterizing all observed interaction patterns.

In Table 3, we report the means and SDs of the three probabilities, along with the 95% confidence intervals (CIs) for the means and the *p*-values from the Shapiro–Wilk (SW) normality tests. The Shapiro–Wilk test results yield *p*-values of .799, .445, and .225, all of which exceed the significance level α=.05. Accordingly, we fail to reject the null hypothesis of normality for all three parameters, indicating no evidence of substantial deviation from normality. The narrow 95% confidence intervals (lengths of .040, .036, and .044) further indicate the precision of the estimated means. We use the sample means as baseline simulation inputs; these descriptive and distributional checks do not establish model validity or account for uncertainty arising from classroom context, the eligibility rule, or unmodeled interaction processes.

**Table 3:** Descriptive statistics and Shapiro–Wilk normality tests for interaction probabilities.

Parameter	#Students	Mean	SD	Min	Max	95% CI	SW-*p*
*P*_rec_	115	0.609	0.109	0.36	0.88	[0.589, 0.629]	.799
*P*_tb_	115	0.451	0.099	0.22	0.68	[0.433, 0.469]	.445
*P*_tg_	117	0.446	0.119	0.15	0.75	[0.424, 0.468]	.225

### Participation and opportunity inequality

The turn-taking model proposed in the Turn-taking modeling section simulates an idealized baseline of turn-taking in triadic groups by retaining selected local turn-transition tendencies while excluding individual-specific factors. In this section, we use the model with the estimated probabilities from the Probability estimation subsection to run participation and opportunity simulations. To reflect typical classroom group activities, we set the number of turns to *n* = 20, 30, 40, 50, 70, 100, 150, 200, 300, 400, 500, where *n* represents the total number of conversational turns occurring in a classroom setting. For each scenario, we perform simulations with 1000 repetitions and then examine the resulting distributions of participation and opportunity inequality measures.

In each simulation, we record the number of speaking turns and the number of addressed turns for each participant. Specifically, for a group 𝒢={A,B,C}, if participant *A* speaks and the entire group 𝒢 is addressed, then *A*’s speaking turn count is incremented by one, while the addressed turn counts of *B* and *C* are simultaneously incremented by one. Under the symmetric baseline, each member has an expected 1/3 share of speaking turns and addressed opportunities, although realized counts may differ. Opportunity shares are calculated relative to the sum of all members’ addressed-turn counts. To quantify inequalities in participation and opportunity, we introduce the following measure. Let $n_{A}$, $n_{B}$, and $n_{C}$ denote the speaking turn counts of participants *A*, *B*, and *C*, respectively. The proportion of speaking turns for each participant is defined as


$$\begin{array}{c} p_{A}=\frac{n_{A}}{n_{A}+n_{B}+n_{C}},\;p_{B}=\frac{n_{B}}{n_{A}+n_{B}+n_{C}},\;p_{C}=\frac{n_{C}}{n_{A}+n_{B}+n_{C}}. \end{array}$$

We measure participation inequality using the SD of these proportions:


$$\begin{array}{c} \gamma_{PART}:=SD(p_{A},p_{B},p_{C})=\sqrt{\frac{(p_{A}-\bar{p}{)}^{2}+(p_{B}-\bar{p}{)}^{2}+(p_{C}-\bar{p}{)}^{2}}{3}},\;\bar{p}=\frac{p_{A}+p_{B}+p_{C}}{3}. \end{array}$$

In the extreme case of maximal inequality, where only one participant speaks (i.e., proportions 1, 0, 0), this measure attains the value $\gamma_{PART}=\frac{\sqrt{2}}{3}\approx 0.4714$. Accordingly, the range of the participation inequality measure is $\gamma_{PART}\in \left[0,\frac{\sqrt{2}}{3}\right]$. Then, we normalize it to [0, 1] by defining ${\tilde{\gamma }}_{PART}:=\frac{3}{\sqrt{2}}\gamma_{PART}$. Analogously, $\gamma_{OPPO}$ is the population SD of each member’s share of all addressed opportunities, and its normalized form is ${\tilde{\gamma }}_{OPPO}=(3/\sqrt{2}\gamma_{OPPO}$.

The raw indices $\gamma_{PART}$ and $\gamma_{OPPO}$ are therefore population SDs of participation and opportunity shares, respectively, and their normalized forms ${\tilde{\gamma }}_{PART}$ and ${\tilde{\gamma }}_{OPPO}$ rescale these indices to [0, 1]. For three shares summing to one, the relation to the Herfindahl–Hirschman concentration index $H=\sum_{i}p_{i}^{2}$ [74] is $\gamma^{2}=(H-1/3)/3$. SD has also been used to describe participation distributions in collaborative-learning studies [38,39].

Here, SD is a practical implementation choice: it is straightforward to compute, and a raw SD of 0.05 represents a root mean squared departure of five percentage points from equal shares. The methodological contribution is the simulation-based calibration procedure rather than a claim that SD is preferable for every study. The same procedure can be applied to other inequality metrics, as illustrated in the metric-comparison analysis below.

The statistics for the various inequality measures are presented in Table 4. As shown in the table, as the number of turns increases, both participation and opportunity inequality measures tend to decrease to zero, indicating that the turn-taking model reflects a situation in which participants achieve nearly equal levels of participation and opportunity. This is intuitive, as a larger number of turns naturally allows opportunities to be distributed more evenly among participants under ideal equitable conditions.

**Table 4:** Simulation results based on 1000 repetitions for triadic groups using SD-based inequality indices. Reported are the mean ($\bar{\mu }$) and 95th percentile (*U*_0.95_) for raw indices and their normalized forms.

#Turns	$\gamma_{PART}$		${\tilde{\gamma }}_{PART}$		$\gamma_{OPPO}$		${\tilde{\gamma }}_{OPPO}$	
	$\bar{\mu }$	*U*_0.95_	$\bar{\mu }$	*U*_0.95_	$\bar{\mu }$	*U*_0.95_	$\bar{\mu }$	*U*_0.95_
20	0.058	0.103	0.124	0.218	0.042	0.084	0.088	0.179
30	0.048	0.098	0.102	0.208	0.034	0.066	0.072	0.139
40	0.041	0.077	0.088	0.164	0.030	0.055	0.063	0.117
50	0.037	0.068	0.079	0.145	0.025	0.050	0.054	0.106
70	0.033	0.064	0.069	0.136	0.022	0.043	0.046	0.091
100	0.027	0.052	0.057	0.111	0.018	0.035	0.039	0.073
150	0.022	0.043	0.046	0.090	0.015	0.028	0.031	0.059
200	0.019	0.038	0.040	0.080	0.013	0.025	0.027	0.053
300	0.015	0.029	0.032	0.063	0.010	0.020	0.022	0.043
400	0.013	0.025	0.027	0.053	0.009	0.017	0.019	0.037
500	0.012	0.022	0.025	0.047	0.008	0.016	0.017	0.033

In addition to the asymptotic behavior of the inequality measures ${\tilde{\gamma }}_{PART}$ and ${\tilde{\gamma }}_{OPPO}$, we examined their behavior during the early stages of turn-taking conversations. Larger fluctuations are observed during approximately the first 50 turns. Furthermore, the simulated distributions of these measures depart significantly from normality (Shapiro–Wilk tests, *p* < .001) and show positive skewness. These observations are illustrated in Fig 3, which illustrates how the simulation results are used to construct a reference value. The temporal simulations indicate that participation inequality fluctuates in early turns, while the distribution across repeated simulations provides the basis for using the 95th percentile as a simulation-based threshold.

**Fig 3:**
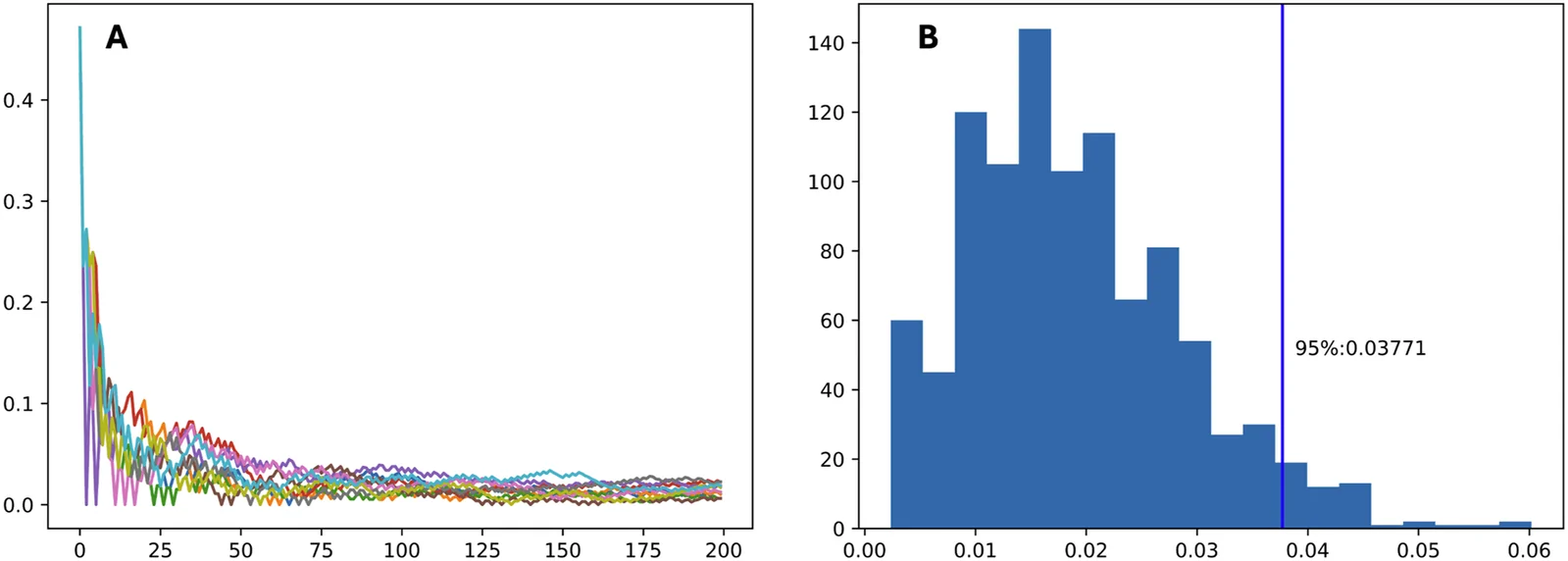
Participation inequality measure $\gamma_{PART}$ for 200 turns and 1000 simulation runs. (A) Temporal evolution of $\gamma_{PART}$ for the first 10 simulations. (B) Histogram of 1000 $\gamma_{PART}$ values up to 200 turns. *Note.* The 95th percentile, *U*_0.95_ = 0.038, indicates that an observed $\gamma_{PART}$ above 0.038 exceeds the range observed in 95% of the idealized baseline simulations and therefore would be flagged as potential participation inequality.

Using the defined inequality measures, we can identify whether a group’s participation or opportunity distribution exceeds the corresponding simulation-based reference value. Specifically, the raw indices $\gamma_{PART}$ and $\gamma_{OPPO}$ capture variability in these shares, and each observed index must be compared with a reference value on the same scale. The simulation results in Table 4 provide a reference for assessment:

**(C) *Within the present calibrated baseline, for a given turn count n, flag potential participation or opportunity inequality when the observed raw index*** $\gamma_{PART}$ ***or*** $\gamma_{OPPO}$***, respectively, is strictly greater than its corresponding raw-scale U***_***0.95***_ ***reference value.***

These reference values are conditional on the model structure and empirical parameter estimates used in the present study and should not be interpreted as universal thresholds for triadic collaboration. Application to other populations, task types, cultural or linguistic contexts, or substantially different interaction conditions requires recalibration using context-appropriate parameter estimates.

The 95th percentile is used as a conventional upper-tail reference point under the specified model, estimated parameters, metric, and turn count, rather than as an educationally validated intervention cutoff. The 90th, 95th, and 97.5th percentiles correspond to nominal upper-tail probabilities of 10%, 5%, and 2.5% under the reference distribution; exact exceedance frequencies may differ because the simulated measures are discrete and the quantiles are estimated. Lower percentile thresholds would flag more episodes, whereas higher thresholds would flag fewer. Selecting an operational warning level therefore requires independent educational evidence and consideration of the consequences of different threshold choices. An observed value above the 95th-percentile reference indicates that it is unusually high relative to the simulated baseline; it does not establish a 95% probability that the group is inequitable or, by itself, justify intervention.

### Empirical triangulation

In this section, we use two empirical triangulation approaches to examine whether the simulation-based criteria identify interpretable participation and opportunity inequality patterns in the empirical dataset described in the Data description subsection. These analyses provide within-sample empirical triangulation rather than independent external validation. Because the teacher-informed benchmark is also defined using cutpoints on participation-share SD, agreement with the simulation criterion should be interpreted as convergence between two operationalizations. The selected discourse cases provide qualitative illustrations of the interaction patterns associated with threshold exceedance.

#### Teacher-informed benchmark comparison.

In practice, the teacher evaluated only participation inequality using an intuitive, teacher-informed benchmark. This benchmark was based on four reference distributions of participation rates in triads: (0.3, 0.3, 0.4) (minor variation, $\gamma_{PART}=0.047$), (0.2, 0.4, 0.4) (the highest participation rate is twice the lowest, $\gamma_{PART}=0.094$), (0.14, 0.43, 0.43) (the highest participation rate is approximately three times the lowest, $\gamma_{PART}=0.137$), and (0, 0.5, 0.5) (one member receiving no turns, $\gamma_{PART}=0.236$). Therefore, the benchmark classified episodes into five ordered levels based on these four benchmarks (see Table 5). For comparison with the simulation-based classification, we further collapsed the teacher-informed benchmark into a binary indicator, in which moderate, substantial, and severe participation inequality were treated as “concerning,” while acceptable variation and slight inequality were treated as “not concerning.”

**Table 5:** Teacher-informed benchmarks for participation inequality.

Group size	Criteria	Level of participation inequality
Triad	$\gamma_{PART}\geq 0.236$	Severe participation inequality
	$0.137\leq \gamma_{PART}<0.236$	Substantial participation inequality
	$0.094\leq \gamma_{PART}<0.137$	Moderate participation inequality
	$0.047\leq \gamma_{PART}<0.094$	Slight participation inequality
	$\gamma_{PART}<0.047$	Within acceptable variation

We then compared the assessment results based on the teacher-informed static benchmark system in Table 6 and the simulation-based turn-sensitive reference criterion in Table 4. For each empirical episode, we matched its observed length to the nearest lower turn count available in the simulation table and used the corresponding raw-scale SD reference value, as printed to three decimals in Table 4. Values strictly greater than the corresponding *U*_0.95_ reference were coded as “flagged for potential inequality.” The normalized indices were included as rescaled descriptive summaries and were not used for classification. Episodes at or below the selected reference were coded as “not flagged”; this designation refers only to their position relative to the simulated reference distribution and does not imply that the episode was educationally equitable.

**Table 6:** Empirical assessment of participation and opportunity inequality and teacher-informed participation benchmark.

#Turns	#Episodes	$\gamma_{PART}$			$\gamma_{OPPO}$	
		Mean (SD)	#Flagged (%)	#Teacher concerning (%)	Mean (SD)	#Flagged (%)
20–29	25	0.114 (0.058)	13 (52.0%)	14 (56.0%)	0.061 (0.042)	7 (28.0%)
30–39	28	0.093 (0.045)	9 (32.1%)	10 (35.7%)	0.050 (0.035)	8 (28.6%)
40–49	35	0.101 (0.062)	19 (54.3%)	15 (42.9%)	0.052 (0.033)	12 (34.3%)
50–69	57	0.096 (0.066)	33 (57.9%)	20 (35.1%)	0.053 (0.047)	24 (42.1%)
70–99	51	0.074 (0.045)	27 (52.9%)	12 (23.5%)	0.046 (0.041)	19 (37.3%)
100–149	36	0.071 (0.051)	20 (55.6%)	6 (16.7%)	0.041 (0.037)	13 (36.1%)
150–199	8	0.067 (0.033)	6 (75.0%)	1 (12.5%)	0.069 (0.030)	8 (100.0%)
200–299	5	0.077 (0.058)	3 (60.0%)	2 (40.0%)	0.065 (0.045)	4 (80.0%)
Total	245	0.089 (0.056)	130 (53.1%)	80 (32.7%)	0.051 (0.040)	95 (38.8%)

Overall, 130 of the 245 episodes were flagged for potential participation inequality, whereas 80 episodes were assessed as concerning by the teacher-informed benchmark (see Table 6). For opportunity inequality, 95 episodes were flagged. Thus, the simulation-based criterion flagged potential participation inequality more frequently than the teacher-informed static benchmark and flagged participation inequality more frequently than opportunity inequality in the observed triadic discussions.

We further examined the relationship between the five-level teacher-informed benchmark and the simulation-based participation classification. Table 7 presents the cross-tabulation. The comparison showed that none of the episodes categorized as acceptable variation by the teacher-informed benchmark were flagged by the simulation-based criterion. In contrast, more than 94% of episodes categorized as moderate, substantial, or severe participation inequality were flagged. The slight inequality category was divided almost evenly between flagged and not flagged episodes, suggesting that slight inequality represents a borderline zone between a static teacher-informed benchmark and the turn-sensitive simulation criterion.

**Table 7:** Cross-tabulation of teacher-informed participation inequality levels and simulation-based participation inequality flagging status.

Teacher-informed level	#Flagged as potential participation inequality (%)	#Total episodes
Within acceptable variation	0 (0.0%)	60
Slight participation inequality	52 (49.5%)	105
Moderate participation inequality	34 (94.4%)	36
Substantial participation inequality	43 (100.0%)	43
Severe participation inequality	1 (100.0%)	1
Total	130 (53.1%)	245

Descriptively, the association between teacher-informed levels and the simulation flag was Cramér’s *V* = .734, and the Spearman correlation between the ordinal teacher level and binary simulation flag was ρ=.727 across 245 episodes. These statistics summarize within-sample association. Because the severe category contains only one episode and episodes repeat students and groups within two classrooms, we do not report independence-based significance tests for these comparisons; formal inferential testing would require an analysis that accounts for this dependence and the sparse categories.

Using the binary teacher-informed benchmark, the simulation-based classification showed 78.0% overall agreement. Relative to this constructed benchmark, descriptive sensitivity was 97.5%, specificity was 68.5%, balanced accuracy was 83.0%, and Cohen’s kappa was κ=.568. These are descriptive agreement statistics rather than estimates of diagnostic accuracy against an independent educational standard. These results suggest that the simulation-based criterion captured most episodes categorized as moderate-or-higher participation inequality by the teacher-informed benchmark, but it also flagged a portion of episodes categorized as slight inequality. Thus, this comparison provides a useful within-sample check on the practical interpretability of the simulation-based participation criterion, while supporting its use as a screening indicator rather than a definitive judgment of educational inequity.

#### Discourse-based triangulation.

We further conducted a limited discourse-based triangulation analysis to examine whether episodes with different simulation-based classifications showed different patterns of participation and opportunity allocation during actual collaborative problem solving.

We drew on the distinction between amplification and attenuation of inequity [8]. Amplification refers to local interactional moves that increase or reproduce unequal access, whereas attenuation refers to moves that reduce, redistribute, or repair unequal access. Shah and Lewis describe participatory equity as involving both opportunities to participate and participation itself, and emphasize that access to the conversational floor is fluid and can shift over time. For this analysis, the unit of qualitative interpretation was an opportunity event, defined as a speaking turn together with its addressee and immediate uptake in subsequent turns. We summarized each selected episode by identifying local moves that amplified or attenuated participation inequality and opportunity inequality. For participation inequality, amplification patterns included sustained speaker dominance, limited contribution by one member, and weak uptake of a less-participating member’s re-entry attempts. Attenuation patterns included successful re-entry, shared production of solutions, and redistribution of task work. For opportunity inequality, amplification patterns included preferential addressing, dyadic enclosure, weak uptake of a peripheral member, and repeated allocation of the task floor to the same peer. Attenuation patterns included whole-group invitations, direct invitations to a less-addressed member, reciprocal uptake, and repair of peripheralization.

To control the scale of the analysis, we selected four episodes from the same task and the same 100–149 turn interval. In this task, students were shown a picture of a snake lying along a brick path and were asked to estimate how many bricks the snake would cover if its body were straightened. The task encouraged groups to explore multiple solution strategies and explain their reasoning. The four cases were chosen to represent the four possible combinations of participation inequality and opportunity inequality classification (see Table 8). This contrastive sampling strategy allowed us to examine whether participation and opportunity classifications captured distinct discourse patterns under comparable task and discussion-length conditions.

**Table 8:** Contrastive cases for discourse-based triangulation.

Case	PART rates	OPPO rates	Turns	$\gamma_{PART}$ flag	$\gamma_{OPPO}$ flag	Main discourse pattern
**G19**			148	Flagged	Flagged	Dyadic enclosure between S28 and S29; S30 peripheral
S28	.480	.478				
S29	.507	.447				
S30	.014	.075				
**G01**			141	Not flagged	Flagged	Speaking turns distributed, but task opportunities concentrated around S32 and S33
S31	.326	.234				
S32	.312	.391				
S33	.362	.375				
**G05**			149	Flagged	Not flagged	S47 spoke most, but opportunities were distributed across all members
S45	.282	.344				
S46	.262	.316				
S47	.456	.340				
**G04**			105	Not flagged	Not flagged	Floor access distributed, although interaction quality was uneven
S42	.371	.377				
S43	.333	.315				
S44	.295	.308				

*Note.* In the Case column, the first line gives the group ID and the following lines list the three students.

The first case, G19(S28/S29/S30), was flagged for both participation inequality and opportunity inequality. This classification was consistent with the qualitative discourse pattern. The task floor became organized mainly as a S28–S29 dyad. S30 made an early contribution by suggesting that the snake occupied approximately five bricks, but this contribution was not developed into a sustained role in the discussion. Later, S28 briefly redirected the floor to S30 by saying that S30 should think about the problem, and near the end the worksheet was handed to S30 to write. However, these moves occurred after most substantive reasoning had already been carried out by S28 and S29. Thus, the episode showed sustained amplification of both participation and opportunity inequality, with only limited late-stage attenuation.

The second case, G01(S31/S32/S33), was not flagged for participation inequality but was flagged for opportunity inequality. This case is important because it demonstrates that opportunity inequality captures a discourse pattern that is not reducible to speaking-turn balance. The discourse suggests that although S31 remained verbally present, the central mathematical floor increasingly became organized around S32 and S33. For example, S32 and S33 repeatedly addressed one another while negotiating measurement, calculation, and solution methods. S31 made several re-entry attempts, including proposing that the group look at the snake’s length, suggesting that the answer might still be six bricks, proposing drawing as a method, and later asking whether S33 had calculated incorrectly. These moves attenuated the imbalance locally, but they did not fully alter the broader pattern in which task-relevant opportunities were concentrated around S32 and S33. Thus, this case supports the interpretive value of the opportunity inequality criterion.

The third case, G05(S45/S46/S47), was flagged for participation inequality but not for opportunity inequality. In this episode, S47 often took a leading role and produced extended reasoning about how to move or straighten the snake. At the same time, S47 repeatedly opened the task floor to both peers by asking S45 why they thought the answer should be five, asking S46 to explain the process, assigning or negotiating writing roles, responding to questions from both peers, and encouraging S46 to continue writing near the end. Therefore, this episode showed participation inequality in production, but not a corresponding concentration of opportunity allocation. This case suggests that a student can speak more than peers while still distributing the conversational floor across the group.

The fourth case, G04(S42/S43/S44), was not flagged for either participation inequality or opportunity inequality. Both speaking turns and addressed opportunities were relatively evenly distributed. The discourse contained some disrespectful language, but the conversational floor was not strongly concentrated around one or two members. All three students contributed to the problem-solving process at different moments: S44 proposed moving part of the snake, S42 suggested rotation and later writing a method, and S43 repeatedly redirected the group back to the task and joined the discussion of possible methods. This case therefore illustrates an important boundary of the proposed metrics. An episode may not be flagged for participation or opportunity inequality while still containing other collaboration-quality problems. The criteria should therefore be interpreted as indicators of distributional imbalance, not as comprehensive measures of participation equity, discourse quality, or educational fairness. Conversely, temporary asymmetry may be compatible with productive collaborative reasoning, for example when one student explains an idea and others listen, question, or build on it. Thus, neither a balanced distribution nor threshold exceedance by itself establishes the educational effectiveness of the collaboration.

Across the four contrastive cases, the discourse patterns were generally consistent with the simulation-based classifications. These findings provide preliminary empirical evidence that the two simulation-based criteria can identify potentially meaningful participation and opportunity inequalities. They also suggest that the two criteria capture related but distinguishable discourse patterns.

### Robustness check using the Gini coefficient

The central contribution of this study is the simulation-based procedure for constructing reference distributions conditional on discussion length and explicit turn-taking assumptions, rather than the use of the SD-based index itself. $\gamma =SD(p_{A},p_{B},p_{C})$ is one convenient and readily interpretable implementation, and we do not argue that it is generally preferable to other measures of participation inequality. Metric selection should follow the research question, participation unit, temporal scale, and intended use of the analysis. Asano et al. [41], for example, show that participation-balance measures can behave differently across participation units and distributions. Peng et al. [10] use a normalized coefficient-of-variation measure based on pairwise turn-taking counts within adaptively identified discussion segments, targeting a different aspect of interaction from the distribution of individual participation shares.

The same simulation procedure can be applied to another metric calculated from the same participation or opportunity shares by computing that metric for each simulated discussion, constructing its reference distribution at each discussion length, and comparing the observed value with the corresponding percentile. Each metric therefore requires its own reference values rather than reuse of the numerical SD cutoffs. For measures that increase with equality rather than inequality, such as some entropy formulations, the corresponding lower tail or a consistently defined inequality transformation would be used. For triadic shares summing to one, the mean is fixed at 1/3, so the coefficient of variation equals three times the population SD; consequently, the coefficient of variation and SD yield identical percentile-based classifications when calculated from the same shares and calibrated consistently. If participation is instead operationalized using different units, such as words or speech duration, the simulation itself would need to represent those units before suitable reference distributions could be generated.

To examine whether the main classification patterns depended on the use of the SD-based indices, we conducted an additional robustness check using the raw Gini coefficient. For each triadic episode, the Gini coefficient was calculated from the three members’ participation or opportunity proportions. We applied the same simulation procedure described in the Participation and opportunity inequality subsection to generate Gini-specific 95th percentile reference thresholds for each turn-count condition (see Table 9). The Gini-based thresholds showed the same turn-sensitive pattern as the standard-deviation-based thresholds. For both participation inequality and opportunity inequality, the Gini-based thresholds showed an overall decreasing pattern as the number of turns increased. This pattern supports the central premise of the simulation approach: an observed level of imbalance should be interpreted relative to the number of turns in the episode.

**Table 9:** Simulation results based on 1,000 repetitions for triadic groups using Gini-based inequality indices. Reported are the simulated mean ($\bar{\mu }$) and 95th percentile threshold (*U*_0.95_) for raw Gini indices and their normalized forms.

Turns	*G*_PART_		${\tilde{G}}_{PART}$		*G*_OPPO_		${\tilde{G}}_{OPPO}$	
	$\bar{\mu }$	*U*_0.95_	$\bar{\mu }$	*U*_0.95_	$\bar{\mu }$	*U*_0.95_	$\bar{\mu }$	*U*_0.95_
20	0.092	0.200	0.139	0.300	0.062	0.123	0.093	0.185
30	0.074	0.133	0.111	0.200	0.053	0.108	0.080	0.162
40	0.064	0.133	0.096	0.200	0.044	0.088	0.066	0.132
50	0.057	0.120	0.085	0.180	0.040	0.081	0.060	0.121
70	0.049	0.095	0.073	0.143	0.033	0.067	0.050	0.100
100	0.042	0.080	0.063	0.120	0.027	0.055	0.041	0.083
150	0.033	0.062	0.049	0.093	0.022	0.042	0.034	0.064
200	0.029	0.057	0.043	0.085	0.020	0.039	0.030	0.059
300	0.023	0.044	0.035	0.067	0.016	0.031	0.024	0.047
400	0.020	0.038	0.030	0.058	0.014	0.028	0.021	0.043
500	0.018	0.036	0.028	0.054	0.013	0.025	0.019	0.037

*Note.* *G*_PART_ and *G*_OPPO_ denote raw Gini-based participation and opportunity inequality indices, respectively. ${\tilde{G}}_{PART}$ and ${\tilde{G}}_{OPPO}$ denote normalized Gini indices, calculated as G×3/2 for triadic groups. *U*_0.95_ refers to the 95th percentile of the simulated reference distribution. Normalized entries were calculated from the full-precision raw values before rounding to three decimals.

We then compared the SD-based and Gini-based results using both raw-score associations and binary classification agreement, applying the same classification rule to the Gini indices with the three-decimal reference values printed in Table 9. As shown in Table 10, the raw SD and Gini values were almost perfectly associated for both participation inequality and opportunity inequality. The two thresholding approaches also produced highly consistent classifications, with over 90% agreement and almost-perfect Cohen’s kappa values for both dimensions.

**Table 10:** Agreement between SD-based and Gini-based classifications.

Dimension	Spearman’s ρ between raw SD and Gini	# SD flagged (%)	# Gini flagged (%)	# Same classification (%)	Cohen’s κ
Participation	.996	130(53.1%)	131(53.5%)	226(92.2%)	.844
Opportunity	.997	95(38.8%)	89(36.3%)	235(95.9%)	.913

*Note.* *N* = 245 episodes. Coefficients summarize within-sample association and agreement; no independent-observation significance claim is made.

Overall, the Gini robustness check shows substantial consistency between the SD-based and Gini-based classifications in this dataset, rather than establishing equivalence among all possible inequality measures. These findings support the robustness and metric-flexibility of the simulation-based thresholding procedure. The Gini comparison therefore illustrates how the same simulation framework can calibrate an alternative metric while leaving metric selection to the needs of the study.

### Stability analysis

In this section, we numerically analyze the stability of the proposed turn-taking model. Specifically, we examine how the three probabilities *P*_rec_, *P*_tb_, and *P*_tg_ affect the inequality measures $\gamma_{PART}$ and $\gamma_{OPPO}$, which are used to flag potential participation and opportunity inequality.

We provide two checks of internal model stability. First, during the simulation of turn-taking conversations, we re-estimate the three probabilities and compare them with the estimates obtained from the empirical dataset (given in Table 3). The re-estimated values and their Shapiro–Wilk *p*-values are reported in Table 11. The results show that the re-estimated probabilities are close to the estimated values of Table 3. Normality-test results vary by parameter and discussion length; the percentile-based inequality criterion does not require normally distributed parameter estimates.

**Table 11:** Descriptive statistics of the three re-estimated parameters using the same 1,000 simulation repetitions per turn-count condition as Table 4; SD denotes the sample SD across repetitions.

# Turns	*P*_rec_		*P*_tb_		*P*_tg_	
	Mean (SD)	SW-*p*	Mean (SD)	SW-*p*	Mean (SD)	SW-*p*
20	0.611 (0.147)	< .001	0.453 (0.113)	< .001	0.439 (0.113)	< .001
30	0.608 (0.124)	0.068	0.448 (0.093)	< .001	0.445 (0.093)	< .001
40	0.607 (0.108)	0.503	0.451 (0.080)	< .001	0.444 (0.078)	< .001
50	0.608 (0.094)	0.036	0.453 (0.071)	< .001	0.441 (0.070)	< .001
70	0.610 (0.081)	0.089	0.448 (0.059)	< .001	0.447 (0.059)	< .001
100	0.607 (0.066)	0.912	0.451 (0.048)	0.005	0.444 (0.047)	< .001
150	0.609 (0.053)	0.607	0.453 (0.042)	0.025	0.444 (0.042)	0.055
200	0.610 (0.047)	0.406	0.449 (0.036)	0.066	0.446 (0.036)	0.135
300	0.608 (0.037)	0.335	0.451 (0.028)	0.085	0.446 (0.029)	0.050
400	0.610 (0.034)	0.842	0.450 (0.024)	0.213	0.447 (0.024)	0.367
500	0.610 (0.030)	0.361	0.451 (0.022)	0.163	0.446 (0.022)	0.247

Second, we observe that the 95th percentiles of the inequality measures are not highly sensitive to changes in the probabilities. In Table 12, we present the values of $U_{0.95}(\gamma_{PART})$ and $U_{0.95}(\gamma_{OPPO})$ for various choices of *P*_rec_, *P*_tb_, and *P*_tg_, which are all close to each other. This suggests that the proposed criterion for assessing inequalities remains consistent across a reasonable range of probability values.

**Table 12:** Sensitivity analysis of inequality measures under perturbations of interaction probabilities (200 turns, 1000 simulations).

*P*_rec_	*P*_tb_	*P*_tg_	$U_{0.95}(\gamma_{PART})$	$U_{0.95}(\gamma_{OPPO})$
0.609	0.451	0.446	0.0377	0.0250
0.589	0.451	0.446	0.0368	0.0245
0.609	0.451	0.424	0.0352	0.0256
0.609	0.433	0.446	0.0358	0.0253
0.609	0.469	0.446	0.0368	0.0247
0.609	0.451	0.468	0.0366	0.0244
0.629	0.451	0.446	0.0371	0.0247

## Discussion and conclusions

### Practical benchmarks for participation and opportunity inequality

This study introduces quantitative benchmarks as preliminary references for interpreting participation and opportunity inequality in triadic group discussions using an agent-based modeling and simulation approach. The empirical parameter estimates underlying these benchmarks were derived from 117 fourth-grade students in two classrooms in mainland China, working in heterogeneous triads on mathematics and reading tasks. Although the stability analysis suggests that the benchmarks are not highly sensitive to reasonable variations in the estimated turn-taking parameters, this internal robustness does not remove the need for external validation across different age groups, cultural contexts, task types, and group sizes before the benchmarks are applied to broader contexts. In the following discussion, we consider how these context-specific reference values may support examination of participation patterns alongside task demands, student roles, and qualitative evidence, rather than serve as general standards of educationally acceptable variation. Conversational norms, classroom culture, age, language, instructional practices, task demands, and group composition may influence the transition probabilities and resulting reference distributions. Application in a new setting should therefore involve assessing whether the model assumptions are appropriate, estimating transition probabilities from comparable local data, regenerating reference distributions for the relevant discussion lengths, and separately evaluating the educational interpretation of those reference values.

As summarized in Table 4, the simulation provides participation and opportunity inequality reference values across discussion lengths from 20 to 500 turns. For instance, a 45-turn discussion with a raw participation SD of 0.080 is compared with the 40-turn reference value of 0.077 under the next-lower-bin rule. This provides an approximation rather than a percentile calibrated specifically for 45 turns. Because 0.080 exceeds the selected 40-turn reference, the episode is flagged for closer examination under this rule. Such exceedance indicates departure from the specified statistical reference and does not by itself establish poor collaboration or a need for intervention.

The reference values are sensitive to the number of turns: as discussions become longer, stricter thresholds are applied. Our findings indicate that all inequality measures tend to approach zero as the number of turns increases. This also highlights the importance of considering the total number of turns when evaluating equity in group discussions. For discussions with fewer turns, disparities in speaking opportunities and participation may be more pronounced due to the limited interaction time. Thus, the statistical interpretation of a given observed imbalance should account for discussion length. Shorter discussions permit greater stochastic variation under the simulated baseline, but this pattern does not imply that different educational standards of acceptable participation should be applied to shorter and longer discussions.

The proposed benchmarks may serve as preliminary references for interpreting participation and opportunity distributions in social group awareness tools or teacher orchestration dashboards [16,19,20]. Because both participation inequality and opportunity inequality fluctuate substantially during the initial phases of discussion, any use of early-stage prompts would require separate evaluation of their timing and educational consequences. The observed early fluctuations suggest a possible direction for research on real-time monitoring, but this study does not establish an effective monitoring-window length. Whether such monitoring helps educators identify meaningful concerns or provide useful support remains to be tested independently. Accordingly, any resulting alerts should be interpreted as screening indicators rather than definitive judgments of inequity. Furthermore, the simulation procedure can be recalibrated for other inequality measures, such as the Gini coefficient, by constructing measure-specific reference distributions rather than reusing the SD cutoffs.

The proposed criteria also offer valuable potential insights for both students and teachers, fostering a better understanding of equal participation and equal participation opportunities. Students, especially young students, tend to perceive themselves as not participating equally if their speaking turns are not identical. Our criteria indicate that demonstrating equality in group discussions with limited time is a challenging task. In practice, neither exact numerical uniformity nor remaining below a statistical reference value is sufficient to establish participation equity or productive collaboration. With reference to these criteria, students can gain a better understanding of their performance regarding participation equity in group discussions. Moreover, the quantitative evaluation criteria offer teachers a valuable tool to screen participation and opportunity inequality issues in group discussions. These reference values may help organize closer examination of participation patterns, but their usefulness for determining the timing or effectiveness of scaffolding has not been established. Although the reference values may support an initial interpretation of group participation patterns, they should be considered alongside contextual knowledge, qualitative observations, task demands, student roles, and the quality of students’ contributions before deciding whether support is needed [1,8]. Balanced turn counts can accompany superficial exchanges, while a useful extended explanation or temporary division of roles can produce unequal counts within productive collaboration. The indices therefore describe structural participation patterns rather than reasoning quality, learning achievement, or the educational value of contributions.

### Implications for understanding participation dynamics

Kapur et al. [39] reported early stabilization of participation inequality in online eleventh-grade triads, while Lämsä et al. [75] observed early lock-in of participation patterns in two five-member undergraduate physics groups. These observations concern persistent participation patterns in empirical collaboration. Our idealized simulations instead show large early fluctuations followed by decreasing participation and opportunity inequality as turn counts increase. This pattern provides a statistical baseline for interpreting discussion length; it does not establish the social or interactional mechanisms responsible for persistent empirical participation patterns.

When the simulation-based reference criterion was applied to the empirical data, participation inequality was flagged more frequently than opportunity inequality overall. These frequencies describe exceedance of the respective simulation-based reference distributions and should not be interpreted as direct evidence of poor collaboration or learning. Whether a flagged episode represents educationally problematic participation requires evidence beyond turn-count distributions. Social awareness tools and communication strategies are possible forms of support [1], but the present analysis does not establish that flagged groups require such support or would benefit from it. Teachers can support equitable groupwork by establishing inclusive participation norms and addressing status differences [46].

Beyond the primary findings concerning quantitative criteria, our empirical data showed no substantial evidence of deviation from normality in the distributions of the three estimated turn-taking parameters (*P*_rec_, *P*_tb_, *P*_tg_). Re-estimation from the simulated discussions produced parameter means close to their generating values, while the Shapiro–Wilk results varied across parameters and discussion lengths. This parameter recovery serves as an internal computational consistency check; it does not establish that the parameter distributions or values are stable across individuals, populations, or interaction contexts. Their use as baseline inputs therefore remains conditional on the empirical calibration data and model assumptions.

These findings provide a context-specific statistical basis for examining participation dynamics, while the educational meaning of departures from that basis remains an empirical question. Future research can evaluate whether reference values generated by this procedure support equitable and productive interaction using independent measures of collaboration quality, student experience, and learning.

## Limitations and future research

This study acknowledges several limitations that warrant attention in future research and practice, including model abstraction, contextual generalizability, the construction of the ideal-equity baseline, and the educational validation of the proposed thresholds.

First, the three parameters used in the simulation, *P*_rec_, *P*_tb_, and *P*_tg_, should be understood as simplified approximations of local turn allocation and addressivity in triadic classroom dialogue, rather than as exhaustive or universal features of human dialogue. Although these parameters are theoretically grounded in turn-taking and participation-shift perspectives, they capture only selected local sequential mechanisms: whether an addressed participant takes up the next turn, whether the next turn is oriented back to the previous speaker, and whether the speaker addresses the whole group rather than a specific individual. Future research should therefore examine whether the proposed parameters remain theoretically and empirically robust across diverse linguistic, cultural, developmental, and instructional contexts, and whether additional parameters are needed to model more complex forms of collaborative interaction.

Second, the empirical parameter estimates and benchmark values used in this study were derived from 117 fourth-grade students in two mainland-China classrooms, working in heterogeneous triads on mathematics and reading tasks. Although the stability analysis suggests that the proposed benchmarks are not highly sensitive to reasonable variations in the estimated parameters, this internal robustness does not establish broad external generalizability. Future research should validate and, where necessary, recalibrate both the parameter estimates and the resulting benchmarks across different age groups, cultural contexts, task types, and instructional settings before applying them to broader collaborative learning contexts. In addition, the study exclusively focuses on triadic groups, while group size is a pivotal factor influencing participation equity. Future research endeavors should aim to establish quantitative evaluation criteria for other common small group sizes, such as four, five, and six-person groups. However, it is important to note that expanding the analysis to larger groups may introduce greater complexity to the simulation model and necessitate additional empirical data for parameter estimation. Within the present sample, parameter estimation included only discussions in which all three members contributed at least 10 speaking turns. This may limit the representativeness of the calibration sample for highly asymmetric interactions. Future work can examine the sensitivity of the parameter estimates and resulting reference distributions to alternative eligibility rules.

Third, this study simulates an ideal-equity reference condition by excluding Type I and Type II personal factors. This design provides a baseline distribution based on local turn allocation and addressee selection when individual and relational asymmetries are not explicitly represented, but it does not capture the full range of participation patterns that may emerge in authentic classroom collaboration. The baseline is less directly applicable when roles or task demands intentionally create asymmetry, and departure from the reference distribution cannot identify whether the observed pattern arises from exclusion, expertise, personality, friendship, social anxiety, or another unmodeled influence. Future research may incorporate Type I and Type II factors into the simulation to model more complex collaboration patterns. Such factors may reflect both initial differences among group members, such as self-concept, verbal confidence, academic status, or peer preference, and history-dependent changes during interaction. For example, a student who repeatedly provides useful explanations may become increasingly likely to be addressed, whereas a student who is repeatedly bypassed may become less likely to take the floor. By systematically varying the strength of these individual, relational, and history-dependent influences, future simulations could generate reference distributions corresponding to different modeled degrees of participation and opportunity inequality. These distributions could support more differentiated reference values, such as slight, moderate, or strong inequality, although their educational interpretation would require independent empirical validation.

Fourth, although the analyses provide a teacher-informed benchmark comparison for participation inequality and discourse-based triangulation for both participation and opportunity inequality, these analyses were conducted within the same empirical dataset used to estimate the model parameters. Therefore, the proposed thresholds should still be interpreted as simulation-based reference criteria rather than fully validated educational standards. No independent classroom dataset in this study links threshold exceedance to poorer collaboration, reduced learning, or the benefit of an intervention. Furthermore, the confidence of these recommended thresholds is based on simulated results, which rely on model assumptions and may not capture all real-world variations in turn-taking behavior. The reference values also depend on estimated transition probabilities and the chosen percentile; internal sensitivity checks do not establish robustness to changes in population, eligibility rules, or educational use. Moreover, the proposed benchmarks are based on the structural distribution of speaking turns and addressed opportunities, rather than on the qualitative content, timing, or interactional function of participants’ contributions. The present coding does not distinguish, for example, whether a turn involved elaboration, questioning, repair, interruption, or a brief acknowledgment. Nor does it capture whether silence reflected disengagement, exclusion, listening, or productive reflection. Therefore, the proposed thresholds are best understood as screening indicators for identifying episodes that warrant closer qualitative or pedagogical examination.

Future research could further validate these quantitative evaluation criteria using independent samples and additional educational evidence, such as expert coding of collaboration quality, teacher judgments, student reports of participation experience, and learning outcome measures. Such work could examine whether episodes flagged by the simulation-based criteria are consistently associated with lower perceived participation access, poorer collaborative quality, or weaker learning gains. In addition, future studies could enrich the measurement of participation equity by incorporating qualitative discourse analysis and more comprehensive content-related quantitative indicators. Recent collaboration analytics research has emphasized the need for process-oriented and theory-guided modelling of individual participation in collaborative learning [76], and has conceptualized individual contributions as multidimensional constructs involving not only the quantity but also the heterogeneity of contributions [77]. Building on this line of work, future research could examine the distribution of reasoning moves, uptake, questioning, explanation quality, epistemic positioning, repair, coordination, and collaborative knowledge-building behaviors. Such extensions would help determine not only whether participation and opportunity are structurally balanced, but also how students use those opportunities to contribute meaningfully to collaborative learning.
