# Supplementary material for: How unequal is unequal? Turn-taking simulation for benchmarking participation and opportunity inequality in small-group collaboration
Source: PLoS One. 2026 Oct 23;21(10):e0361205. doi: 10.1371/journal.pone.0361205 (PMC13693943; doi:10.1371/journal.pone.0361205)
Supplement: Supplementary file 1 [file pone.0361205.s004.pdf]

Response to Reviewers

June 25, 2026

General Response

Dear Editor Dr. Indriyanto and Reviewers,

We sincerely thank you for granting us the opportunity to revise our manuscript (PONE-D-26-11209). We are grateful for your careful reading of our manuscript and for your valuable comments and suggestions. We have diligently addressed each of the comments. In the revised manuscript, you will find the changes in blue. We hope that our revisions meet your expectations, and we remain open to addressing any additional concerns or suggestions from you.

In this response letter, for convenience, reviewer comments are set in *italics* and introduced by **red headings**, while our responses are introduced with **blue headings**.

Response to Reviewer 1

Reviewer Comment 1:

> *First, while the modeling framework is technically interesting, the study relies heavily on simulation to derive its main conclusions. The empirical dataset appears to be used primarily for parameter estimation rather than for rigorous validation of the proposed thresholds. This creates a disconnect between the real-world data and the claims made. The manuscript would be significantly strengthened by incorporating stronger empirical validation, for example by testing whether the proposed inequality thresholds predict meaningful educational outcomes or observable interactional imbalances in actual classroom data.*

Response: Thank you for this important comment. In the revised manuscript, we largely revised original Section 4.4 to triangulate the simulation-based classifications with empirical classroom evidence, per your valuable suggestions.

Specifically, we added two forms of empirical triangulation. First, for participation inequality, we added a *teacher-informed benchmark comparison* (Section 4.4.1). The teacher-informed benchmark classified participation inequality into five ordered levels: within acceptable variation, slight participation inequality, moderate participation inequality, substantial participation inequality, and severe participation inequality based on four reference distributions of participation rates (see Table 5). For comparison with the simulation-based classification, we found that none of the episodes categorized as acceptable variation by the teacher-informed benchmark were flagged by the simulation-based criterion. In contrast, more than 94% of episodes categorized as moderate, substantial, or severe participation inequality were flagged. The slight inequality category was divided almost evenly between flagged and not flagged episodes, suggesting that slight inequality

represents a borderline zone between a static teacher-informed benchmark and the turn-sensitive simulation criterion (see Table 7).

We also added inferential and agreement statistics for this comparison. The association between the five-level teacher-informed benchmark and the simulation-based participation classification was significant and strong, $\chi^2(4) = 132.03$, $p < .001$, Cramér's $V = 0.734$. Because the teacher-informed benchmark was ordinal, we also conducted a Spearman rank-order correlation, which showed a strong positive association between teacher-informed imbalance level and simulation classification, $\rho = .727$, $p < .001$, $n = 245$.

We collapsed teacher-informed five levels into a binary benchmark in which moderate, substantial, and severe participation inequality were treated as "concerning," while acceptable variation and slight inequality were treated as "not concerning." Using the binary teacher-informed benchmark, the simulation-based classification showed 78.0% overall agreement, 97.5% sensitivity, 68.5% specificity, 83.0% balanced accuracy, and Cohen's $\kappa = .568$. These results provide empirical support that the participation criterion corresponds to a practitioner-informed benchmark, while also showing that the simulation-based criterion should be interpreted as a screening indicator rather than as a definitive educational judgment.

Second, because no teacher-informed benchmark was available for opportunity inequality, we added a *discourse-based triangulation analysis* for both participation inequality and opportunity inequality (see Section 4.4.2). To control the scope of the analysis, we selected four episodes from the same task and the same 100–149 turn interval, representing the four possible combinations of simulation classification: both participation and opportunity inequality flagged, only opportunity inequality flagged, only participation inequality flagged, and neither flagged. We analyzed whether the discourse showed amplification or attenuation of participation and opportunity inequality. This contrastive analysis showed that the case flagged for both dimensions displayed both speaker dominance and restricted opportunity allocation; the case flagged only for opportunity inequality showed balanced speaking turns but unequal access to the task-relevant floor; the case flagged only for participation inequality showed speaker dominance but distributed addressee opportunities; and the case not flagged for either dimension showed relatively distributed participation and opportunity. These findings provide preliminary empirical evidence that the two simulation-based criteria can identify potentially meaningful participation and opportunity inequalities. They also suggest that the two criteria capture related but distinguishable discourse patterns. At the same time, we interpret these findings as empirical triangulation rather than definitive educational validation, given their within-sample nature and limited educational-outcome evidence.

In response to reviewers' shared concern, we further revised the interpretation of the thresholds throughout the manuscript. We no longer present the thresholds as fully validated educational criteria. Instead, we describe them more cautiously as *simulation-based reference thresholds* or *screening indicators*. Exceeding a threshold is now interpreted as evidence that an episode shows a level of participation or opportunity imbalance beyond the expected range under the ideal simulation. Conversely, episodes not exceeding the threshold are described as "not flagged," rather than "fair" or "equitable." We believe these revisions substantially reduce the previous disconnect between the simulation model and the empirical classroom data.

Changes in Manuscript: Please see Section 4.4 *Empirical Triangulation*, pp. 14–18, of the revised manuscript. We added two forms of empirical validation: a comparison with teacher-informed benchmarks (Section 4.4.1) and triangulation through qualitative discourse analysis of four representative cases (Section 4.4.2).

Reviewer Comment 2:

Second, the decision to exclude Type I and Type II individual factors from the simulation in order to construct "ideal equity" conditions is understandable from a modeling perspective, yet it raises concerns about ecological validity. Participation in real classrooms is inherently shaped by individual, social, and cultural variables. The current model may therefore oversimplify the phenomenon it aims to represent. The authors should more explicitly discuss the implications of this abstraction and clearly delimit the scope of their claims.

Response: Thank you for this important comment. We agree that participation in real classroom collaboration is shaped by individual, social, and cultural factors, and that the exclusion of Type I and Type II factors needs to be interpreted carefully. Our intention was not to suggest that these factors are irrelevant to classroom collaboration. Rather, the exclusion of these factors was a modeling abstraction used to construct an idealized baseline condition.

The purpose of this baseline model is to estimate the range of participation and opportunity variation that may arise from local turn allocation and addressivity when systematic individual or social advantages are held constant. In other words, the model asks a narrower question: under equal-status assumptions, how much imbalance in speaking turns and addressed opportunities can still occur because dialogue is sequentially organized rather than purely random? This baseline can then be used as a reference point for identifying episodes that show potential structural imbalance beyond what would be expected under the simulated ideal-equitable condition.

We also agree that the model should not be interpreted as a full representation of authentic classroom collaboration. More complex participation patterns may emerge when local dialogue structures interact with asymmetric and changing Type I and Type II factors. For example, students may enter the discussion with different levels of self-concept, verbal confidence, academic status, or peer preference, and these differences may affect their initial likelihood of taking the floor or being addressed. These probabilities may also change during interaction: a student who repeatedly provides useful explanations may become increasingly likely to be addressed, whereas a student who is repeatedly bypassed may become less likely to take the floor. Thus, future models incorporating individual, relational, and dynamic Type I and Type II factors would be better suited for simulating more realistic classroom collaboration patterns and for developing more detailed benchmarks for different degrees of participation or opportunity inequality.

Accordingly, we now interpret the proposed thresholds as simulation-based reference criteria for screening potential structural imbalance, rather than as definitive judgments of educational inequity or comprehensive indicators of collaboration quality.

Changes in Manuscript: Please see Section 3.3 *Idealized equitable baseline simulation*, p. 10. Here we further clarified our purpose of excluding individual and social factors is to construct a constrained baseline model that suppresses systematic individual advantages or disadvantages while retaining minimal turn-level structures of multiparty dialogue.

Please also see Section 6 *Limitations and Future Research*, p. 22. In this section (the "First" limitation), we further discuss the limitations of the baseline model and suggest that future research combine local turn-taking structures with Type I and Type II factors to model more complex discussions and provide more detailed interpretive guidance.

Reviewer Comment 3:

Third, although the statistical procedures used are generally appropriate, the analytical rigor remains limited for a high-impact journal. The study relies largely on descriptive statistics, normality tests, and simulation percentiles. There is no use of inferential

statistical modeling, no reporting of effect sizes, and no comparison with alternative inequality measures such as the Gini coefficient. Providing a stronger statistical justification for the chosen metrics and triangulating results with alternative indices would enhance the robustness of the findings.

Response: Thank you for your valuable suggestions. In the revised manuscript, we strengthened the analysis by clarifying the inferential logic of the simulation-based framework, adding effect-size and agreement statistics, incorporating empirical triangulation, and comparing the SD-based results with Gini-based results.

Our main contribution is to develop a principled statistical framework for evaluating whether observed participation or opportunity imbalance exceeds a model-based reference range while explicitly accounting for the number of turns. Specifically, we model the turn-taking process through three probabilities: P_{rec}, P_{tb}, and P_{tg}. After estimating these parameters from real classroom data, we repeatedly simulate the full generative process to construct an empirical distribution of inequality measures. With a sufficiently large number of simulations, such as 1,000 repetitions, this distribution provides an approximation to the induced sampling distribution under the model.

We then use the 95th percentile of this empirical distribution as a reference threshold. Under the simulated ideal-equity turn-taking process, approximately 95% of simulated inequality values fall below this threshold. Therefore, if an observed conversation yields an inequality value exceeding the corresponding threshold, we interpret it as model-based evidence that the observed imbalance is beyond the expected range under the reference model.

Overall, this procedure constitutes a simulation-based statistical inference framework: we observe limited data and use a generative model to infer properties of an underlying latent process. This setting differs from classical approaches such as regression, since there is no trustworthy response variable indicating "inequality" versus "no inequality." Instead, inference is mostly carried out indirectly through the distribution of a derived statistic, which motivates our proposed simulation methodology.

In response to the concern about empirical validation (see our response to your Comment 1), we added a teacher-informed benchmark comparison for participation inequality which brings in more advanced statistics. The association between the five-level teacher-informed benchmark and the simulation-based classification was significant, $\chi^2(4) = 132.03$, $p < .001$, with a large effect size, Cramér's $V = 0.734$. Because the teacher-informed benchmark was ordinal, we also conducted a Spearman rank-order correlation, which showed a strong positive association between teacher-informed imbalance level and simulation classification, $\rho = .727$, $p < .001$, $n = 245$. Using a binary teacher-informed benchmark, the simulation-based classification showed 78.0% overall agreement, 97.5% sensitivity, 68.5% specificity, 83.0% balanced accuracy, and Cohen's $\kappa = .568$. These analyses provide additional inferential and effect-size evidence.

We also added discourse-based triangulation for both participation inequality and opportunity inequality. Four contrastive cases were selected from the same task and the same turn-count interval. The qualitative analysis showed that these classifications corresponded to distinguishable discourse patterns, including speaker dominance, unequal floor allocation, their overlap, and their absence. This addition strengthens the empirical interpretation of the simulation-based criteria and clarifies that participation inequality and opportunity inequality capture related but non-redundant aspects of collaborative discourse.

Finally, following your suggestion, we added a robustness check using the Gini coefficient (see Section 4.5). We quantified participation inequality and opporunity inequality using Gini values and generated Gini-specific 95th percentile thresholds using the same simulation procedure. The raw SD and Gini values were almost perfectly associated for both participation and opportunity

inequality, $\rho = .997$, $p < .001$. The SD-based and Gini-based classifications also showed high agreement: 92.2% agreement for participation inequality, Cohen's $\kappa = .844$, $p < .001$, and 95.9% agreement for opportunity inequality, Cohen's $\kappa = .913$, $p < .001$. These findings indicate that the empirical classification patterns were robust to the use of Gini as an alternative inequality index.

Together, these revisions provide stronger statistical justification, effect-size evidence, empirical triangulation, and robustness testing. They also clarify that the primary contribution of the study is not the exclusive use of SD, but the simulation-based framework for generating turn-sensitive reference thresholds that can be applied to different inequality indices.

Changes in Manuscript: Please see Section 4.4 *Empirical Triangulation*, pp. 14–18, of the revised manuscript. We added two forms of empirical validation and brought in more inferential statistics.

Please see Section 4.5 *Robustness Check Using the Gini Coefficient*, pp. 18–19. In this section, we triangulated the SD-based results with an alternative index Gini-coefficient.

Reviewer Comment 4:

> *Fourth, the issue of data availability is a significant concern. The manuscript states that the data cannot be publicly shared and are only available upon request. This does not align with PLOS data transparency requirements. The authors need to either provide a publicly accessible, de-identified dataset or offer a much clearer and policy-compliant justification for the restriction.*

Response: Thank you for raising this important concern. In response to this comment, we have revised our data-sharing plan.

Specifically, we have deposited a publicly accessible reproducibility package on Zenodo: `https://doi.org/10.5281/zenodo.20855572`. This package includes the de-identified empirical analytic dataset, the author-generated simulation code, and the simulation output files needed to reproduce the quantitative findings reported in the manuscript. The de-identified empirical dataset includes turn-taking sequences and participation and opportunity metrics for each episode. The repository also includes the Python scripts used to conduct the turn-taking simulations and generate benchmark thresholds.

At the same time, we have not publicly released the raw classroom audio recordings, full transcripts, or individual-level student background and grouping information because the study involved minor children in small classroom groups. These materials may contain direct or indirect identifiers, including voice information, classroom-specific references, academic grouping information, peer relationship information, and sociometric nomination data. Public release of these raw materials could compromise participant confidentiality, even after removal of direct identifiers. Requests for access to restricted materials may be directed to the Human Research Ethics Committee, The University of Hong Kong, via Research Services, The Registry, The University of Hong Kong, at rssdata@hku.hk. Access requests will be reviewed in accordance with participant consent, institutional ethics approval, institutional policy, and applicable privacy regulations.

Changes in Manuscript: Please see the updated Data Availability Statement

Reviewer Comment 5:

> *Fifth, the generalizability of the findings is limited. The sample consists of fourth-grade students from a specific educational context, and the tasks are relatively narrow in scope. While this is acknowledged in the limitations section, the discussion tends to present the*

proposed benchmarks as broadly applicable. The authors should moderate their claims and more carefully contextualize the applicability of their model.

Response: Thank you for this important comment. We agree that the generalizability of the proposed benchmarks should be more carefully bounded. In the revised manuscript, we moderated claims about the applicability of the benchmarks and avoided presenting them as broadly generalizable educational criteria. Instead, we now describe them more cautiously as simulation-based reference thresholds for interpreting participation and opportunity distributions in triadic group discussions similar to the empirical context of this study.

At the same time, we would like to clarify that the manuscript includes a stability analysis showing that the proposed benchmarks are not highly sensitive to reasonable variations in the estimated turn-taking parameters. This analysis supports the internal robustness of the simulation-based thresholds within the specified model. However, we agree that such robustness does not, by itself, establish broad external generalizability. Therefore, we further clarified that the parameter estimates were derived from fourth-grade students in a specific educational and cultural context, working on a limited set of mathematics and reading tasks. We also expanded the limitations section to emphasize that future research should validate and, where necessary, recalibrate the benchmarks across different age groups, cultural contexts, task types, and group sizes before broader application.

Changes in Manuscript: Please see Section 5.1 *Practical Benchmarks for Participation and Opportunity Inequality*, pp. 20–21. At the very beginning of the discussion, we clarified that the benchmarks should be interpreted within the boundary conditions of the present study, including the sample of fourth-grade students, the specific cultural and educational context, the task types, and the triadic group structure.

Throughout the Discussion section, we also moderated relevant claims to avoid presenting the proposed benchmarks as general or definitive references.

Please also see Section 6 *Limitations and Future Research*, p. 22. We restructured the Limitation section to more clearly contextualize the generalizability of the proposed benchmarks (the "Second" limitation).

Reviewer Comment 6:

Finally, minor revisions are needed in language and style. While the manuscript is generally clear, some sections, particularly the methodology and literature review, could be streamlined to improve readability. There are occasional redundancies and minor grammatical inconsistencies that should be corrected.

Response: We revised the manuscript to improve language clarity, readability, and overall flow. We streamlined the literature review and methodology sections by reducing redundant expressions and clarifying several lengthy or ambiguous sentences. We also reviewed the manuscript for minor grammatical inconsistencies, formatting issues, and wording problems, and made corrections throughout the text.

Changes in Manuscript: These revisions can be found throughout the revised manuscript, especially in Sections 2.2–2.3, 2.5, 3, 4.3, 4.4, 5, and 6.

Response to Reviewer 2

Reviewer Comment 1:

> *Major Concern 1. The theoretical justification for the three dialogue parameters is insufficient. The entire simulation framework relies heavily on three parameters: Prec, Ptb, and Ptg. The manuscript argues that these parameters represent "features intrinsic to human dialogue" (p. 7). However, the theoretical basis for selecting only these three interaction mechanisms is not sufficiently justified.*
>
> *Three-person collaborative interaction is substantially more complex than the proposed model suggests. Real collaborative dialogue includes many additional interactional processes, such as interruption, coalition formation, dominance accumulation, repair, epistemic positioning, silence, emotional reciprocity, and long-term interactional dependencies. The current model reduces interaction dynamics primarily to immediate turn-transition probabilities.*
>
> *While simplification is understandable in simulation research, the manuscript needs to more clearly explain why these three parameters are theoretically sufficient to represent equitable turn-taking dynamics. At present, the selection of these parameters appears somewhat arbitrary and under-theorized.*
>
> *I strongly recommend that the authors provide a more substantial grounding in conversation analysis, dialogic interaction theory, or turn-taking literature to justify the modeling assumptions.*

Response: Thank you for this important and constructive comment. In response, we revised the manuscript to provide a more explicit theoretical grounding for the three dialogue parameters and to use more cautious language in describing their scope.

Specifically, we now frame the three parameters as a parsimonious representation of local turn allocation and addressivity in triadic group dialogue, rather than as exhaustive or universal features of human dialogue (see Section 3.2). We strengthened the theoretical rationale for these parameters by drawing more explicitly on conversation analysis, turn-taking theory, and small-group interaction research. Together, these parameters allow the simulation to preserve basic sequential structures of multiparty dialogue rather than treating equitable participation as independent random selection of speakers and addressees.

We also clarified why the model focuses on these three parameters (see Section 3.3). The purpose of the simulation is not to reproduce the full complexity of authentic classroom collaboration, but to construct an ideal-equity reference condition. Under this condition, systematic individual and social advantages are held constant, while selected local structures of turn allocation and addressivity are retained. In this sense, the model addresses a narrower question: how much variation in participation and opportunity distributions can arise from local turn-taking structure alone?

At the same time, we added a clearer limitation regarding model abstraction (see Section 6). We now acknowledge that real collaborative dialogue involves many additional processes, including interruption, repair, epistemic positioning, silence, emotional reciprocity, coalition-like alignment, dominance accumulation, and long-term interactional dependencies. Although P_{rec}, P_{tb}, and P_{tg} are theoretically grounded in turn-taking and participation-shift perspectives, they capture only selected local sequential mechanisms. Future research should therefore examine whether these parameters remain theoretically and empirically robust across diverse linguistic, cultural, developmental, and instructional contexts, and whether additional parameters are needed to model more complex forms of collaborative interaction.

Changes in Manuscript: Please see Section 3.2, *Local Sequential Parameters in Triadic Dialogue*, pp. 8–9. We restructured this section to provide a more explicit theoretical justification for the three dialogue parameters and to clarify their meanings. We also revised the language to describe these parameters more cautiously as simplified approximations of local turn allocation and addressivity in triadic classroom dialogue, rather than as exhaustive or universal features of human dialogue.

Please see Section 3.3, *Idealized Equitable Baseline Simulation*, p. 10. We clarified that the purpose of the constrained baseline model is to suppress systematic individual advantages or disadvantages while retaining minimal turn-level structures of multiparty dialogue.

Please also see Section 6, *Limitations and Future Research*, p. 22. We expanded the limitation noting that the current model captures selected local sequential mechanisms and that future research should examine whether additional parameters are needed to model more complex forms of collaborative interaction (the "First" limitation).

Reviewer Comment 2:

> *Major Concern 2. The manuscript makes overly strong claims about "intrinsic dialogue features." A major conceptual issue is that the three parameters are estimated from real classroom discussions that already contain participation inequalities, social dynamics, status effects, and interpersonal asymmetries. However, the manuscript later treats these parameters as representing idealized "intrinsic" properties of human dialogue and uses them to simulate "ideally equitable" group interaction.*
>
> *This creates an important conceptual tension.*
>
> *The empirical classroom data used to estimate Prec, Ptb, and Ptg likely already reflect the influence of academic status, friendship, confidence, anxiety, dominance, and other social factors. Averaging across groups does not necessarily remove these structural inequalities. Therefore, it is difficult to conclude that the resulting parameters represent purely dialogue-intrinsic mechanisms.*
>
> *The manuscript would benefit from a more cautious interpretation. Instead of framing these parameters as universal intrinsic dialogue features, the authors may consider describing them as simplified approximations of turn-taking tendencies observed in their dataset.*

Response: Thank you for identifying this important conceptual issue. We agree that the original manuscript used overly strong language in describing P_{rec}, P_{tb}, and P_{tg} as "intrinsic" dialogue features.

In the revised manuscript, we adopt a more cautious interpretation and strengthen the theoretical justification for these parameters (also see our response to Comment 1). Following your suggestion, we now describe the three parameters as simplified, empirically estimated approximations of local turn-taking tendencies observed in the present triadic classroom dataset. They are used to preserve basic turn allocation and addressivity structures in the simulation, but they are no longer presented as universal or purely intrinsic properties of human dialogue.

We agree that averaging across groups cannot fully remove the influence of academic status, friendship, confidence, anxiety, dominance, or other structural inequalities in the original data. This concern also motivated the stability analysis included in the previous manuscript. The stability analysis showed that the proposed benchmarks are not highly sensitive to reasonable variations in the estimated turn-taking parameters, which supports the internal robustness of the simulation-based thresholds within the specified model. However, such internal robustness does not, by itself,

establish broad external generalizability or demonstrate that the parameters are context-free properties of human dialogue.

Therefore, we further emphasized in the Discussion section that the parameter estimates were derived from fourth-grade students in a specific educational and cultural context working on a limited set of mathematics and reading tasks, and we clarified the boundary conditions for applying the benchmarks. We also expanded the Limitations section to state that future research should validate and, where necessary, recalibrate both the parameter estimates and the resulting benchmarks across different age groups, cultural contexts, task types, instructional settings, and group sizes before broader application.

Changes in Manuscript: Throughout the manuscript, especially Section 3.2–3.3, pp. 8–10, we replaced the phrase "intrinsic features of human dialogue" with more cautious wording, such as "local sequential parameters," "turn allocation and addressivity," and "turn-taking tendencies observed in the present dataset."

Please see Section 5.1 *Practical Benchmarks for Participation and Opportunity Inequality*, pp. 20-21. At the beginning of the Discussion section, we clarified the contextual scope of the empirical parameter estimates and the resulting benchmarks.

Please also see Section 6, *Limitations and Future Research* (the "Second" limitation), p. 22, where we suggest that these parameters should be recalibrated and validated across different learning settings, cultural contexts, age groups, group sizes, subject domains, and task types before the benchmarks are applied more broadly.

Reviewer Comment 3:

> *Major Concern 3. The interaction model is overly simplified and ignores longer-term interaction dynamics. The proposed simulation model effectively functions as a first-order Markov process, where the next conversational action depends primarily on the immediately preceding turn. However, collaborative interaction is often shaped by cumulative history and emergent group dynamics.*
>
> *For example, many interactional phenomena relevant to participation equity emerge over time, including:*
>
> ** dominance stabilization, * coalition formation, * role differentiation, * increasing withdrawal, * evolving participation norms, * interactional repair processes.*
>
> *Ironically, the manuscript itself discusses "lock-in" effects in participation dynamics later in the discussion section, yet the simulation model does not meaningfully incorporate history-dependent mechanisms.*
>
> *The authors should acknowledge more explicitly that the current model captures only a highly simplified local structure of turn-taking rather than the broader temporal dynamics of collaboration.*

Response: Thank you for this insightful comment. We agree that the current simulation model captures a simplified local structure of turn-taking and does not fully incorporate the broader temporal dynamics of collaboration. In particular, the model primarily uses the immediately preceding turn to determine the next speaker and addressee, and therefore does not directly model cumulative history or feedback processes through which earlier participation patterns shape later interaction.

In the revised manuscript, we have clarified that this simplification was intentional for the purpose of constructing an ideal-equity reference model (see Section 3.3). The model was designed

to estimate how much variation in participation and opportunity distribution may arise from local turn allocation and addressivity alone, while holding systematic individual, relational, or cultural factors constant. Thus, the model should not be interpreted as a full temporal model of collaborative interaction. Rather, it provides a simple baseline against which observed participation and opportunity distributions can be compared.

We also revised the manuscript to distinguish between stable dialogue-structural parameters and more dynamic individual or relational processes (see Section 3.2). Specifically, P_{rec}, P_{tb}, and P_{tg} are now described as local parameters capturing recipient uptake, sequential return, and open-floor addressing. Longer-term participation patterns, such as dominance stabilization, coalition-like alignment, role differentiation, and increasing withdrawal, may emerge when these local structures operate together with asymmetric and changing Type I and Type II factors. For example, a student who repeatedly provides useful explanations may become increasingly likely to be addressed, whereas a student who is repeatedly bypassed may become less likely to take the floor. Such processes would require dynamic speaker-selection and addressee-selection probabilities, rather than fixed dialogue-structural parameters alone.

Changes in Manuscript: Please see Section 3.2, *Local Sequential Parameters in Triadic Dialogue*, p. 9. We expanded the discussion of how local turn-taking structures may interact with asymmetric and dynamic Type I and Type II factors to produce more complex participation patterns.

Please see Section 3.3, *Idealized Equitable Baseline Simulation*, p. 10. We clarified that the purpose of the constrained baseline model is to intentionally suppress systematic individual advantages or disadvantages while retaining minimal turn-level structures of multiparty dialogue.

Reviewer Comment 4:

> *Major Concern 4. The operationalization of "equity" is too narrow. The manuscript operationalizes participation equity primarily through the standard deviation of speaking-turn distributions. While this provides a convenient quantitative metric, it risks oversimplifying the educational meaning of equity.*
>
> *The literature on collaborative learning has long recognized that equal speaking frequency does not necessarily imply equitable participation. Students may contribute differently through:*
>
> ** active listening, * reflection, * coordination, * summarization, * questioning, * epistemic mediation, * or productive silence.*
>
> *The manuscript briefly acknowledges this issue in the literature review, but the later modeling framework still equates equity largely with quantitative balance in turn counts.*
>
> *This limitation should be discussed much more explicitly. Otherwise, the manuscript risks implying that equitable collaboration can be sufficiently reduced to distributional equality of speaking turns.*

Response: Thank you for this important comment. We agree that participation equity cannot be fully reduced to equal speaking frequency or balanced turn counts. Equal numbers of turns do not necessarily mean that students have participated equitably, and unequal numbers of turns do not necessarily indicate that the interaction was educationally inequitable.

In the original manuscript, we acknowledged that prior studies have often focused on promoting equal participation among individuals or demographic groups, and we introduced opportunity distribution as an additional aspect of participation equity informed by Shah and Lewis (2019) and

our previous study (Hu et al., 2024; see Section 2.5). However, we agree that the manuscript did not sufficiently carry this conceptual caution into the later modeling framework and interpretation of the benchmarks.

In response, we revised the manuscript to clarify that the proposed benchmarks capture only the structural distribution of speaking turns and addressed opportunities. They should therefore be interpreted as preliminary screening indicators rather than as comprehensive measures of participation equity. Specifically, exceeding the proposed threshold indicates that an episode shows participation inequality or opportunity inequality beyond the expected range under the simulated ideal-equity reference model. It does not, by itself, establish that the interaction was educationally inequitable. For example, a group may show unequal speaking turns because one student is productively listening, coordinating nonverbal work, or reflecting before contributing. Conversely, a group may show similar turn counts while still displaying inequitable interaction if some turns are dismissive, low-quality, off-task, or fail to take up others' ideas. These distinctions require discourse-content analysis and qualitative interpretation beyond the turn-count framework used in the present study.

We also added this issue as a major limitation. The revised manuscript now states that the proposed benchmarks are based on the structural distribution of speaking turns and addressed opportunities, rather than on the qualitative content, timing, or interactional function of participants' contributions. The present coding does not distinguish, for example, whether a turn involved elaboration, questioning, repair, interruption, or a brief acknowledgment. Nor does it capture whether silence reflected disengagement, exclusion, listening, or productive reflection. Therefore, the proposed thresholds are best understood as screening indicators for identifying episodes that warrant closer qualitative or pedagogical examination.

Changes in Manuscript: Please see Section 6, *Limitations and Future Research* (the "Fourth" limitation), p. 23. We explicitly acknowledged that the current study focuses on the structural distribution of speaking turns and addressed opportunities, and therefore provides limited evidence regarding the broader construct of participation equity.

Throughout the manuscript, we reviewed the use of key terms, including "participation equity," "participation equality," and "opportunity equality," to clarify the conceptual distinction between participation equity as a broader theoretical construct and the current study's narrower analytical focus on participation equality and opportunity equality.

Throughout the manuscript, especially in Section 5.1 *Practical Benchmarks for Participation and Opportunity Inequality*, pp. 20–21, we revised the language to frame the proposed benchmarks as preliminary screening indicators for identifying potential participation and opportunity inequality, rather than as definitive judgments of educational inequity.

Reviewer Comment 5:

> *Major Concern 5. The proposed thresholds lack educational validation. The manuscript derives participation inequality thresholds from simulation percentiles and proposes that groups exceeding the 95th percentile threshold can be considered inequitable with "95% confidence."*
>
> *However, these thresholds are statistically simulated rather than educationally validated.*
>
> *At present, the manuscript does not demonstrate whether the proposed thresholds meaningfully correspond to:*
>
> ** teacher judgments of inequity, * expert coding of collaboration quality, * student ex-*

*periences, * or learning outcomes.*

As a result, the current thresholds should be interpreted more cautiously as simulation-based reference distributions rather than validated educational criteria.

I strongly encourage the authors to discuss this limitation more carefully and avoid overstating the practical interpretability of the thresholds.

Response: Thank you for this important comment. We agree that the proposed thresholds should not be interpreted as fully validated educational criteria. In the original manuscript, the phrase "95% confidence" may have suggested a stronger educational interpretation than warranted. In the revised manuscript, we clarified that this confidence level refers to the simulated ideal-equity reference distribution, not to a definitive judgment about participation or opportunity inequality.

Accordingly, we revised the interpretation of the thresholds throughout the manuscript. We now describe them more cautiously as *simulation-based reference thresholds* or *screening indicators*. Exceeding a threshold is now interpreted as evidence that an episode shows a level of participation or opportunity imbalance beyond the expected range under the simulated ideal-equity reference model. It does not, by itself, establish that the interaction involves participation or opportunity inequality issues. Conversely, episodes not exceeding the threshold are described as "not flagged," rather than "fair".

To strengthen the educational interpretability of the thresholds, we also added empirical triangulation analyses, which are described in detail in our response to Major Concern 6. These analyses provide preliminary empirical support that the simulation-based classifications correspond to practitioner-informed and discourse-based patterns in classroom data. However, we continue to interpret them cautiously as preliminary validation rather than as definitive educational validation. We also expanded the Limitations section to emphasize that future research should further validate the thresholds using independent expert coding, teacher judgments, student reports of participation experience, and learning outcome measures.

Changes in Manuscript: Please see Section 4.4, *Empirical Triangulation*, pp. 14–18, of the revised manuscript. We added empirical triangulation analyses to examine whether the simulation-based classifications correspond to teacher-informed benchmarks and qualitative discourse patterns.

Throughout the manuscript, especially in Section 4.4, *Empirical Triangulation*, pp. 14–18, and Section 5.1, *Practical Benchmarks for Participation and Opportunity Inequality*, pp. 20–21, we refined the language to describe the proposed thresholds more cautiously as preliminary simulation-based references or screening indicators for identifying potential participation or opportunity inequality that warrants further pedagogical or qualitative examination.

Please also see Section 6, *Limitations and Future Research*, p. 22. We explicitly emphasized that the proposed benchmarks require further educational validation using independent samples and additional evidence, such as expert coding, teacher judgments, student reports, and learning outcome measures.

Reviewer Comment 6:

Major Concern 6. The manuscript would benefit from stronger qualitative or empirical triangulation. Relatedly, the paper currently relies almost entirely on computational simulation and descriptive statistics. Additional validation approaches would substantially strengthen the contribution.

For example, the authors could:

** compare simulated inequity classifications with teacher evaluations, * analyze whether identified inequitable groups show poorer collaboration quality, * or conduct qualitative analyses of groups near versus above threshold values.*

Without such triangulation, the educational significance of the simulation outputs remains somewhat uncertain.

In summary, I believe this manuscript addresses an important problem and proposes an interesting computational approach for operationalizing participation equity. However, the current version relies on several strong theoretical assumptions that are insufficiently justified, and the educational interpretation of the proposed thresholds remains underdeveloped. I therefore recommend major revision before the manuscript can be considered for publication.

Response: Thank you for this constructive suggestion. We agree that the educational significance of the simulation-based thresholds requires stronger empirical triangulation. In response, we substantially revised Section 4.4 to triangulate the simulation-based classifications with two forms of empirical classroom evidence.

First, for participation inequality, we added a *teacher-informed benchmark comparison* (Section 4.4.1). The teacher-informed benchmark classified participation inequality into five ordered levels: within acceptable variation, slight participation inequality, moderate participation inequality, substantial participation inequality, and severe participation inequality, based on four reference distributions of participation rates. When compared with the simulation-based classification, none of the episodes categorized as within acceptable variation by the teacher-informed benchmark were identified by the simulation-based criterion. In contrast, more than 94% of episodes categorized as moderate, substantial, or severe participation inequality were identified. The slight inequality category was divided almost evenly between identified and non-identified episodes, suggesting that slight inequality represents a borderline zone between a static teacher-informed benchmark and the turn-sensitive simulation criterion.

We also added inferential and agreement statistics for this comparison. The association between the five-level teacher-informed benchmark and the simulation-based participation classification was significant and strong, $\chi^2(4) = 132.03$, $p < .001$, Cramér's $V = 0.734$. Because the teacher-informed benchmark was ordinal, we also conducted a Spearman rank-order correlation, which showed a strong positive association between teacher-informed inequality level and simulation classification, $\rho = .727$, $p < .001$, $n = 245$. After collapsing the teacher-informed benchmark into a binary classification, the simulation-based classification showed 78.0% overall agreement, 97.5% sensitivity, 68.5% specificity, 83.0% balanced accuracy, and Cohen's $\kappa = .568$.

Second, because no teacher-informed benchmark was available for opportunity inequality, we added a *discourse-based triangulation analysis* for both participation inequality and opportunity inequality (Section 4.4.2). To control the scope of the analysis, we selected four episodes from the same task and the same 100–149 turn interval, representing the four possible combinations of simulation classification: both participation and opportunity inequality identified, only opportunity inequality identified, only participation inequality identified, and neither identified. This contrastive analysis showed that the case identified for both dimensions displayed both speaker dominance and restricted opportunity allocation; the case identified only for opportunity inequality showed relatively distributed speaking turns but unequal access to the task-relevant floor; the case identified only for participation inequality showed speaker dominance but distributed addressee opportunities; and the case not identified for either dimension showed relatively distributed participation and opportunity.

Together, these analyses provide preliminary empirical evidence that the two simulation-based criteria can identify potentially meaningful participation and opportunity inequality patterns in authentic classroom discourse. They also suggest that the two criteria capture related but distinguishable discourse patterns. At the same time, we interpret these findings cautiously as empirical triangulation rather than definitive educational validation, because the analyses were conducted within the same dataset used to estimate the model parameters and provide limited evidence regarding broader educational outcomes. Further validation with independent samples and additional educational indicators remains necessary.

Changes in Manuscript: Please see Section 4.4, *Empirical Triangulation*, pp. 14–18, of the revised manuscript. We added two forms of empirical triangulation: a comparison with teacher-informed benchmarks for participation inequality (Section 4.4.1) and qualitative discourse-based triangulation of four representative cases for both participation and opportunity inequality (Section 4.4.2). We also added inferential and agreement statistics to evaluate the correspondence between teacher-informed and simulation-based classifications.

Response to Reviewer 3

Reviewer Comment 1:

> *1. Limited Empirical Validation The most critical limitation is the weak link between simulation outputs and real-world data: • The empirical dataset is used mainly for parameter estimation, not for validating the proposed thresholds. • There is no inferential testing demonstrating that the thresholds meaningfully detect inequality in authentic classroom settings. • The claim that 66*Strengthen empirical validation by: • Testing whether thresholds predict observable outcomes (e.g., learning gains, engagement) • Comparing flagged vs. non-flagged groups using inferential statistics • Including qualitative validation (e.g., discourse analysis)*

Response: Thank you for this important comment. We agree that the original manuscript relied too heavily on simulation outputs and did not provide sufficient empirical evidence connecting the proposed thresholds to authentic classroom interaction. In the revised manuscript, we strengthened the empirical triangulation in two ways and moderated our interpretation of the classification results.

First, for participation inequality, we added a *teacher-informed benchmark comparison* (Section 4.4.1). The teacher-informed benchmark classified participation inequality into five ordered levels: within acceptable variation, slight participation inequality, moderate participation inequality, substantial participation inequality, and severe participation inequality, based on four reference distributions of participation rates. When compared with the simulation-based classification, none of the episodes categorized as within acceptable variation by the teacher-informed benchmark were identified by the simulation-based criterion. In contrast, more than 94% of episodes categorized as moderate, substantial, or severe participation inequality were identified. The slight inequality category was divided almost evenly between identified and non-identified episodes, suggesting that slight inequality represents a borderline zone between a static teacher-informed benchmark and the turn-sensitive simulation criterion.

We also added inferential and agreement statistics for this comparison. The association between the five-level teacher-informed benchmark and the simulation-based participation classification was

significant and strong, $\chi^2(4) = 132.03$, $p < .001$, Cramér's $V = 0.734$. Because the teacher-informed benchmark was ordinal, we also conducted a Spearman rank-order correlation, which showed a strong positive association between teacher-informed inequality level and simulation classification, $\rho = .727$, $p < .001$, $n = 245$. After collapsing the teacher-informed benchmark into a binary classification, the simulation-based classification showed 78.0% overall agreement, 97.5% sensitivity, 68.5% specificity, 83.0% balanced accuracy, and Cohen's $\kappa = .568$.

Second, because no teacher-informed benchmark was available for opportunity inequality, we added a *discourse-based triangulation analysis* for both participation inequality and opportunity inequality (Section 4.4.2). To control the scope of the analysis, we selected four episodes from the same task and the same 100–149 turn interval, representing the four possible combinations of simulation classification: both participation and opportunity inequality identified, only opportunity inequality identified, only participation inequality identified, and neither identified. This contrastive analysis showed that the case identified for both dimensions displayed both speaker dominance and restricted opportunity allocation; the case identified only for opportunity inequality showed relatively distributed speaking turns but unequal access to the task-relevant floor; the case identified only for participation inequality showed speaker dominance but distributed addressee opportunities; and the case not identified for either dimension showed relatively distributed participation and opportunity.

Together, these analyses provide preliminary empirical evidence that the two simulation-based criteria can identify potentially meaningful participation and opportunity inequality patterns in authentic classroom discourse. They also suggest that the two criteria capture related but distinguishable discourse patterns. At the same time, we interpret these findings cautiously as empirical triangulation rather than definitive educational validation, because the analyses were conducted within the same dataset used to estimate the model parameters and provide limited evidence regarding broader educational outcomes. Further validation with independent samples and additional educational indicators remains necessary.

We also revised the wording of the empirical results. Rather than claiming that a certain percentage of groups "show inequality" as a validated educational conclusion, we now describe these groups as flagged by the simulation-based reference thresholds as showing potential participation or opportunity inequality that warrants closer qualitative or pedagogical examination. Similarly, groups below the threshold are described as "not flagged" by the simulation-based criterion, rather than as definitively fair or equitable. This revision better reflects the status of the proposed thresholds as screening indicators rather than fully validated educational standards.

Although learning-gain or problem-solving score prediction would provide valuable external validation, we did not include it as a central analysis in this revision because the proposed thresholds are episode-level discourse indicators, whereas learning outcomes are shaped by many additional student-, task-, and classroom-level factors. We therefore focused on empirical triangulation evidence more directly aligned with the construct measured by the thresholds: teacher-informed judgments of participation inequality and qualitative discourse patterns of participation and opportunity allocation. We now identify outcome-based validation, student-experience data, independent expert coding, and independent-sample replication as important directions for future research.

Changes in Manuscript: Please see Section 4.4 *Empirical Triangulation*, pp. 14–18, of the revised manuscript. We added two forms of empirical validation and brought in more inferential statistics.

Throughout the manuscript, especially in Section 4.4, *Empirical Triangulation*, pp. 14–18, and Section 5.1, *Practical Benchmarks for Participation and Opportunity Inequality*, pp. 20–21, we refined the language to describe the proposed thresholds more cautiously as preliminary simulation-based references or screening indicators for identifying potential participation or opportunity in-

equality that warrants further pedagogical or qualitative examination.

Please also see Section 6, *Limitations and Future Research*, p. 22. We explicitly emphasized that the proposed benchmarks require further educational validation using independent samples and additional evidence, such as expert coding, teacher judgments, student reports, and learning outcome measures.

Reviewer Comment 2:

> *2 Ecological Validity of the Model The simulation assumes "ideal equity" by excluding Individual characteristics (e.g., ability, personality) and Social dynamics (e.g., status, friendship) While methodologically convenient, this assumption oversimplifies real classroom interactions and limits applicability of the derived benchmarks *You need to provide stronger justification for excluding these variables, clearly delimit the scope of conclusions and consider incorporating variability in future modeling*

Response: Thank you for this important comment. We agree that participation in real classroom collaboration is shaped by individual, social, and cultural factors, and that the exclusion of Type I and Type II factors needs to be interpreted carefully. Our intention was not to suggest that these factors are irrelevant to classroom collaboration. Rather, the exclusion of these factors was a modeling abstraction used to construct an idealized baseline condition.

The purpose of this baseline model is to estimate the range of participation and opportunity variation that may arise from local turn allocation and addressivity when systematic individual or social advantages are held constant. In other words, the model asks a narrower question: under equal-status assumptions, how much imbalance in speaking turns and addressed opportunities can still occur because dialogue is sequentially organized rather than purely random? This baseline can then be used as a reference point for identifying episodes that show potential structural imbalance beyond what would be expected under the simulated ideal-equitable condition.

We also agree that the model should not be interpreted as a full representation of authentic classroom collaboration. More complex participation patterns may emerge when local dialogue structures interact with asymmetric and changing Type I and Type II factors. For example, students may enter the discussion with different levels of self-concept, verbal confidence, academic status, or peer preference, and these differences may affect their initial likelihood of taking the floor or being addressed. These probabilities may also change during interaction: a student who repeatedly provides useful explanations may become increasingly likely to be addressed, whereas a student who is repeatedly bypassed may become less likely to take the floor. Thus, future models incorporating individual, relational, and dynamic Type I and Type II factors would be better suited for simulating more realistic classroom collaboration patterns and for developing more detailed benchmarks for different degrees of participation or opportunity inequality.

Accordingly, we now interpret the proposed thresholds as simulation-based reference criteria for screening potential structural imbalance, rather than as definitive judgments of educational inequity or comprehensive indicators of collaboration quality.

Changes in Manuscript: Please see Section 3.3 *Idealized equitable baseline simulation*, p. 10. Here we further clarified our purpose of excluding individual and social factors is to construct a constrained baseline model that suppresses systematic individual advantages or disadvantages while retaining minimal turn-level structures of multiparty dialogue.

Please also see Section 6 *Limitations and Future Research*, pp. 22–23. In this section, we further discuss the limitations of the baseline model and suggest that future research combine local turn-

taking structures with Type I and Type II factors to model more complex discussions and provide more detailed interpretive guidance.

Reviewer Comment 3:

> *3 Statistical Rigor and Analytical Depth The statistical analysis is technically correct but insufficiently rigorous: • Over-reliance on descriptive statistics and simulation percentiles • No inferential modeling (e.g., regression, multilevel analysis) • No effect sizes • No comparison with alternative inequality metrics (e.g., Gini coefficient) *You should introduce inferential statistical analyses, justify metric choice more robustly and compare results across multiple inequality indices*

Response: Thank you for this valuable comment. Following your suggestions, we have strengthened the statistical analysis in three ways. First, we clarified the inferential logic of the simulation-based thresholding framework. The goal of the proposed analysis is not merely to report descriptive inequality values, but to determine whether an observed participation or opportunity imbalance exceeds the expected range under a simulated ideal-equity reference model while explicitly accounting for the number of turns. Specifically, after estimating the turn-taking parameters P_{rec}, P_{tb}, and P_{tg} from empirical classroom data, we repeatedly simulate triadic turn-taking processes to construct empirical reference distributions of the inequality measures. The 95th percentile of each distribution is then used as a turn-sensitive screening threshold. Thus, the method constitutes a simulation-based inference approach rather than a descriptive percentile summary alone.

We also considered your suggestion regarding regression or multilevel modeling. We did not introduce regression as the primary analytical framework because there is no independent gold-standard outcome variable indicating whether each episode is truly unequal or not. Instead, the central inferential task is to compare each observed inequality statistic with the distribution expected under the simulated ideal-equity model.

Second, to strengthen the empirical interpretation of the criteria, we added inferential tests comparing the simulation-based classification with an external teacher-informed benchmark for participation inequality (see Section 4.4.1). The association between the five-level teacher-informed benchmark and the simulation-based participation classification was statistically significant, $\chi^2(4) = 132.03$, $p < .001$, with a large effect size, Cramer's $V = .734$. Because the teacher-informed benchmark was ordinal, we also conducted a Spearman rank-order correlation, which showed a strong positive association between teacher-informed imbalance level and simulation-based classification, $\rho = .727$, $p < .001$, $n = 245$. Using a binary teacher-informed benchmark, the simulation-based classification showed 78.0% overall agreement, 97.5% sensitivity, 68.5% specificity, 83.0% balanced accuracy, and Cohen's $\kappa = .568$. These analyses add inferential, effect-size, and agreement evidence that was absent from the original manuscript.

Third, following your suggestion, we added a robustness check using the Gini coefficient as an alternative inequality metric (see Section 4.5). We recalculated participation inequality and opportunity inequality using Gini values and generated Gini-specific 95th percentile thresholds using the same simulation procedure. The raw SD and Gini values were almost perfectly associated for both participation inequality and opportunity inequality, $\rho = .997$, $p < .001$. The SD-based and Gini-based classifications also showed high agreement: 92.2% agreement for participation inequality, Cohen's $\kappa = .844$, $p < .001$, and 95.9% agreement for opportunity inequality, Cohen's $\kappa = .913$, $p < .001$. These findings indicate that the empirical classification patterns were robust to the use of Gini as an alternative inequality index. In addition, we strengthened the justification for retaining the SD-based index as the primary measure. Although other inequality measures, such

as Gini and Theil indices, can be used, the SD-based index directly represents dispersion from equal distribution among group members and can be applied consistently to both participation and opportunity distributions. We also clarify that the primary contribution of the study is not the exclusive use of SD, but the simulation-based framework for generating turn-sensitive reference thresholds that can be extended to other inequality measures.

Changes in Manuscript: Please see Section 4.4 *Empirical Triangulation*, pp. 14–18, of the revised manuscript. We added two forms of empirical validation and brought in more inferential statistics.

Please see Section 4.5 *Robustness Check Using the Gini Coefficient*, pp. 18–19. In this section, we triangulated the SD-based results with an alternative index Gini-coefficient.

Reviewer Comment 4:

> *4 Data Availability and Transparency The manuscript states that data are not publicly available. Provide a publicly accessible, de-identified dataset, or offer a clear, policy-compliant justification with institutional restrictions*

Response: Thank you for raising this important concern. In response to this comment, we have revised our data-sharing plan.

Specifically, we have deposited a publicly accessible reproducibility package on Zenodo: `https://doi.org/10.5281/zenodo.20855572`. This package includes the de-identified empirical analytic dataset, the author-generated simulation code, and the simulation output files needed to reproduce the quantitative findings reported in the manuscript. The de-identified empirical dataset includes turn-taking sequences and participation and opportunity metrics for each episode. The repository also includes the Python scripts used to conduct the turn-taking simulations and generate benchmark thresholds.

At the same time, we have not publicly released the raw classroom audio recordings, full transcripts, or individual-level student background and grouping information because the study involved minor children in small classroom groups. These materials may contain direct or indirect identifiers, including voice information, classroom-specific references, academic grouping information, peer relationship information, and sociometric nomination data. Public release of these raw materials could compromise participant confidentiality, even after removal of direct identifiers. Requests for access to restricted materials may be directed to the Human Research Ethics Committee, The University of Hong Kong, via Research Services, The Registry, The University of Hong Kong, at rssdata@hku.hk. Access requests will be reviewed in accordance with participant consent, institutional ethics approval, institutional policy, and applicable privacy regulations.

Changes in Manuscript: Please see the updated Data Availability Statement

Reviewer Comment 5:

> *5 Generalizability The study is limited to fourth-grade students, a single cultural context (China) and specific task types. Yet, the discussion occasionally implies broader applicability. *You should use moderate claims, clearly define boundary conditions and expand discussion of cultural/task variability*

Response: Thank you for your comments. We agree that the boundary conditions of the study should be made more explicit. In the revised manuscript, we moderated claims about the applicability of the benchmarks and avoided presenting them as broadly generalizable educational

criteria. Instead, we now describe them more cautiously as simulation-based reference thresholds for interpreting participation and opportunity distributions in triadic group discussions similar to the empirical context of this study.

At the same time, we would like to clarify that the manuscript includes a stability analysis showing that the proposed benchmarks are not highly sensitive to reasonable variations in the estimated turn-taking parameters. This analysis supports the internal robustness of the simulation-based thresholds within the specified model. However, we agree that such robustness does not, by itself, establish broad external generalizability. Therefore, we further clarified that the parameter estimates were derived from fourth-grade students in a specific educational and cultural context, working on a limited set of mathematics and reading tasks. We also expanded the limitations section to emphasize that future research should validate and, where necessary, recalibrate the benchmarks across different age groups, cultural contexts, task types, and group sizes before broader application.

Changes in Manuscript: Please see Section 5.1 *Practical Benchmarks for Participation and Opportunity Inequality*, pp. 20–21. At the very beginning of the discussion, we clarified that the benchmarks should be interpreted within the boundary conditions of the present study, including the sample of fourth-grade students, the specific cultural and educational context, the task types, and the triadic group structure.

Throughout the Discussion section, we also moderated relevant claims to avoid presenting the proposed benchmarks as general or definitive references.

Please also see Section 6 *Limitations and Future Research*, pp. 22–23. We restructured the Limitation section to more clearly contextualize the generalizability of the proposed benchmarks (the "Second" limitation).

Reviewer Comment 6:

> *6 Writing and Style is generally clear, but some sections are verbose and repetitive. Methodology could be streamlined for readability and there are some minor grammatical inconsistencies*

Response: Thank you for your comment. We revised the manuscript to improve readability, reduce repetition, and address minor language and style issues. We streamlined parts of the literature review and methodology sections by reducing redundant explanations, clarifying lengthy sentences, and improving the flow of the methodological description. We also revised the explanation of the simulation procedure and the idealized baseline model to make methodological logic easier to follow. In addition, we reviewed the manuscript for minor grammatical inconsistencies, wording issues, and formatting problems, and made corrections throughout the text.

Changes in Manuscript: These revisions can be found throughout the revised manuscript, especially in Sections 2.2–2.3, 2.5, 3, 4.3, 4.4, 5, and 6.

Reviewer Comment 7:

> *7 Literature Review could engage more critically with competing metrics. You should include more recent (2023–2025) SSCI studies*

Response: Thank you for your comment. We have incorporated recent studies on participation analytics and participation-balance measures, and provided a more critical discussion of competing quantitative metrics. In the revised manuscript, we also clarify that participation can be

operationalized through different units and formulas, and that these metrics are not directly interchangeable because they capture different aspects of participation distribution.

Changes in Manuscript: Please see Section 2.3, *Absence of Benchmarks for Quantitative Metrics*, p. 4.

Reviewer Comment 8:

> *8 Ensure consistent use of "inequality" vs. "inequity", "participation" vs. "contribution"*

Response: Thank you for your comment. We have carefully reviewed the manuscript and refined the use of these key terms to ensure more consistent usage.

In the revised manuscript, "participation equity" refers to the broader educational and theoretical construct concerning fair access to and engagement in collaborative interaction, while "participation equality" and "opportunity equality" refer to the two analytical dimensions examined in this study. These two dimensions are further operationalized through turn-based indicators, namely γ_{PART} and γ_{OPPO} in this study.

We also clarified that these analytical dimensions should not be conflated with the broader construct of participation equity. A case identified by the turn-based indicators as showing potential participation or opportunity inequality does not necessarily constitute educational inequity. Conversely, participation equality and opportunity equality, as measured in this study, do not by themselves ensure participation equity, because participation equity also depends on the qualitative content, timing, and interactional function of students' contributions.

Accordingly, as noted in our previous responses, we revised the manuscript to interpret the simulation-based thresholds more cautiously as preliminary references or screening indicators for identifying potential participation or opportunity inequality, rather than as definitive educational criteria. When an observed value exceeds the simulation-based reference threshold, we describe the case as showing potential participation or opportunity inequality that warrants closer qualitative or pedagogical examination. We avoid using "inequity" when referring to the quantitative thresholds, because the proposed criteria identify structural distributional patterns rather than make definitive judgments about educational inequity.

We also reviewed the use of "participation" and "contribution". In the revised manuscript, we use "participation" when referring to the turn-based measure used in the simulation and empirical analyses. We use "contribution" only when discussing the broader qualitative content or function of students' input, such as reasoning, questioning, explanation, or coordination. This distinction helps clarify that the present study focuses on the structural distribution of turns and opportunities, rather than the full qualitative value of students' contributions.

Changes in Manuscript: Please see Section 2 *Literature Review*, pp. 2–7. We revised the Literature Review to frame it under the broader conceptual construct of participation equity. We then specified the study's focus on the two analytical dimensions in the Section 2.5 *Present Study* (p. 6). In Section 2.5, we also further clarified the relationship between "participation (in)equity", "participation (in)equality", and "opportunity (in)equality".

Throughout the manuscript, especially in Section 5.1 *Practical Benchmarks for Participation and Opportunity Inequality* (pp. 20–21), we revised the language to frame the proposed benchmarks as preliminary screening indicators for identifying potential participation and opportunity inequality, rather than as definitive judgments of educational inequity.

Please see Section 6, *Limitations and Future Research* (the "Fourth" limitation), p. 23. We explicitly acknowledged that the current study focuses on the structural distribution of speaking turns and addressed opportunities, and therefore provides limited evidence regarding the broader construct of participation equity.

Throughout the manuscript, especially in sections 2.5, 4.3, 4.4 and 5.1, we have refined the usage of these terms to improve conceptual consistency.

Reviewer Comment 9:

> *9 Figures are useful but require clearer captions and more explicit interpretation in the text*

Response: Thank you for your comment. We revised the captions of Figs. 1–3 and added more explicit interpretations in the main text to clarify the role of each figure in the manuscript. Specifically, we clarified that Fig. 1 illustrates the interpretive challenge of using visual participation feedback without benchmarks; Fig. 2 distinguishes actual participation from opportunities to participate; and Fig. 3 explains how the simulation results support the construction of a 95th-percentile reference value.

Changes in Manuscript: Please see Fig. 1 and the more explicit interpretation in Section 1 *Introduction* (pp. 1–2).

Please see Fig. 2 and the more explicit interpretation in Section 2.5 *Present Study* (p. 6).

Please see Fig. 3 and the more explicit interpretation in Section 4.3 *Participation and Opportunity Inequality* (pp. 13–14).

Conclusion

We again thank you for your insightful comments and constructive suggestions.
