# Supplementary material for: How unequal is unequal? Turn-taking simulation for benchmarking participation and opportunity inequality in small-group collaboration
Source: PLoS One. 2026 Oct 23;21(10):e0361205. doi: 10.1371/journal.pone.0361205 (PMC13693943; doi:10.1371/journal.pone.0361205)
Supplement: Supplementary file 2 [file pone.0361205.s005.pdf]

Response to Reviewers

September 24, 2026

General Response

Dear Editor Dr. Indriyanto and Reviewer,

Please consider the second revision of our manuscript (PONE-D-26-11209R1). We are grateful for your careful reading of our manuscript and for your valuable comments and suggestions. We have diligently addressed each of the comments. In the revised manuscript, you will find the changes in blue. We hope that our revisions meet your expectations, and we remain open to addressing any additional concerns or suggestions from you.

In this response letter, for convenience, reviewer comments are set in *italics* and introduced by **red headings**, while our responses are introduced with **blue headings**.

Response to Reviewer 1

Reviewer Comment 1:

> *The most significant concern relates to the generalizability of the proposed benchmark values. The simulation parameters are estimated from a relatively specific empirical context consisting of 117 fourth-grade students from only two classrooms in mainland China who worked in heterogeneous triads on mathematics and reading tasks. Nevertheless, throughout the manuscript the proposed benchmark values are presented in a manner that could easily be interpreted as generally applicable reference thresholds. Since conversational norms, classroom culture, instructional practices, age, task characteristics, and cultural background are all likely to influence the estimated transition probabilities, the proposed thresholds should be presented more cautiously. Rather than describing them as benchmarks for participation inequality in general, they should be explicitly characterized as preliminary reference values derived from one educational context. The discussion would benefit from a more explicit consideration of how these benchmark values may vary across educational settings and how future studies might recalibrate the model for different populations and collaborative environments.*

Response: Thank you for this important comment. We agree that the numerical reference values must be interpreted within their calibration context. The revised study aim describes them as preliminary and context-specific, and Section 4.3 states that they are conditional on the model structure and empirical parameter estimates. The Discussion and Limitations identify the sample of 117 fourth-grade students in two mainland-China classrooms working in heterogeneous triads on mathematics and reading tasks.

We have also clarified what transfer to another setting would require. Section 5.1 explains that conversational norms, classroom culture, age, language, instructional practices, task demands and group composition may affect the transition probabilities. Researchers would need to assess the model assumptions, estimate parameters from comparable local data, regenerate reference distributions for the relevant discussion lengths, and evaluate their educational interpretation separately. The Limitations section explicitly distinguishes internal parameter robustness from broad external generalizability.

Changes in Manuscript: 1. Abstract, p. 1 "Using the standard deviation of these rates, the study establishes preliminary, context-specific simulation-based reference values for identifying departures from an idealized triadic turn-taking baseline...These reference values describe structural participation patterns rather than collaboration quality and require recalibration and independent validation before application in other educational settings."

2. Section 2.5, p. 6, lines 235–237 "This study seeks to generate preliminary, context-specific reference values for these two metrics that distinguish observed imbalances from variation under the specified turn-taking baseline."

3. Section 4.3, p. 14, lines 551–555 "These reference values are conditional on the model structure and empirical parameter estimates used in the present study and should not be interpreted as universal thresholds for triadic collaboration. Application to other populations, task types, cultural or linguistic contexts, or substantially different interaction conditions requires recalibration using context-appropriate parameter estimates."

4. Section 5.1, p. 22, lines 798–804 "Conversational norms, classroom culture, age, language, instructional practices, task demands, and group composition may influence the transition probabilities and resulting reference distributions. Application in a new setting should therefore involve assessing whether the model assumptions are appropriate, estimating transition probabilities from comparable local data, regenerating reference distributions for the relevant discussion lengths, and separately evaluating the educational interpretation of those reference values."

5. Section 6, p. 24, lines 906–911 "Second, the empirical parameter estimates and benchmark values used in this study were derived from 117 fourth-grade students in two mainland-China classrooms, working in heterogeneous triads on mathematics and reading tasks. Although the stability analysis suggests that the proposed benchmarks are not highly sensitive to reasonable variations in the estimated parameters, this internal robustness does not establish broad external generalizability."

Reviewer Comment 2:

> *Although the authors have expanded the methodological justification for selecting the standard deviation as the primary inequality measure, I remain unconvinced that the manuscript sufficiently explains why this metric should be preferred over alternative measures that are already well established in the literature, such as the Gini coefficient, entropy-based indices, or the coefficient of variation. The literature review appropriately acknowledges these alternatives; however, the manuscript ultimately adopts the standard deviation without fully demonstrating why it is particularly advantageous for simulation-based benchmarking. A more explicit discussion of its statistical properties, interpretability, computational simplicity, and suitability for educational applications would strengthen the methodological argument. Even a brief supplementary comparison illustrating that similar benchmark patterns emerge when alternative inequality indices are used would substantially reinforce confidence in the proposed approach.*

Response: Thank you for this important comment. We have clarified that the main contribution of the study is the simulation procedure for constructing reference distributions conditional on discussion length and explicit turn-taking assumptions, rather than the use of SD itself. SD is used as a practical implementation because it is symmetric, straightforward to compute, and readily interpretable on the participation-share scale; for example, a raw population SD of 0.05 represents a root mean squared departure of five percentage points from equal shares. We do not claim that SD is generally superior to established alternatives. Section 4.5 now states explicitly that metric selection should follow the research question, participation unit, temporal scale, and intended use.

We also revisited Peng et al. and Asano et al. and revised the literature review and Section 4.5 to distinguish their measurement choices. Peng et al. use a normalized coefficient-of-variation measure based on pairwise turn-taking counts within adaptively identified discussion segments, which targets a different aspect of interaction from individual participation shares. Asano et al. demonstrate that participation-balance measures can behave differently across participation units and distributions. These studies reinforce our revised framing that metric choice should be aligned with the construct and analytical purpose rather than assuming that one measure is universally preferable.

The revised manuscript further explains how the simulation procedure can be transferred to other metrics. A different metric can be calculated for each simulated discussion, producing its own reference distribution and percentile values at each discussion length. Thus, the procedure is metric-flexible, but the numerical SD reference values are not directly transferable to other scales. For measures that increase with equality rather than inequality, the corresponding lower tail or an inequality transformation would be used. Likewise, measures based on different participation units, such as words or speech duration, would require the simulator to represent those units. For the same three shares summing to one, the coefficient of variation equals three times the population SD, so consistently calibrated percentile classifications coincide; this mathematical equivalence does not extend to Peng et al.'s different operationalization.

Finally, we retained and clarified the Gini comparison added in the first revision (Tables 9 and 10). The SD- and Gini-based results show similar dependence on discussion length and substantial classification agreement in the present dataset. We interpret this as supporting the robustness and metric flexibility of the simulation-based calibration procedure within the present context, rather than as evidence of equivalence among all inequality measures. We therefore have not added an entropy comparison or claimed that the findings are metric-independent.

Changes in Manuscript: 1. Section 4.3, p. 13, lines 521–526 "Here, SD is a practical implementation choice: it is straightforward to compute, and a raw SD of 0.05 represents a root mean squared departure of five percentage points from equal shares. The methodological contribution is the simulation-based calibration procedure rather than a claim that SD is preferable for every study. The same procedure can be applied to other inequality metrics, as illustrated in the metric-comparison analysis below."

2. Section 4.5, p. 19, lines 718–720 "The central contribution of this study is the simulation-based procedure for constructing reference distributions conditional on discussion length and explicit turn-taking assumptions, rather than the use of the SD-based index itself."

3. Section 4.5, p. 19, lines 729–736 "The same simulation procedure can be applied to another metric calculated from the same participation or opportunity shares by computing that metric for each simulated discussion, constructing its reference distribution at each discussion length, and comparing the observed value with the corresponding percentile. Each metric therefore requires its own reference values rather than reuse of the numerical SD cutoffs. For measures that increase with equality rather than inequality, such as some entropy formulations, the corresponding lower

tail or a consistently defined inequality transformation would be used."

4. Section 4.5, p. 20, lines 760–765 "Overall, the Gini robustness check shows substantial consistency between the SD-based and Gini-based classifications in this dataset, rather than establishing equivalence among all possible inequality measures. These findings support the robustness and metric-flexibility of the simulation-based thresholding procedure. The Gini comparison therefore illustrates how the same simulation framework can calibrate an alternative metric while leaving metric selection to the needs of the study."

Reviewer Comment 3:

> *The simulation framework itself is elegant but necessarily simplified. The authors correctly explain that the purpose is to establish an idealized baseline by excluding individual characteristics such as academic status, personality, friendship, social anxiety, and gender. While this simplification is understandable, the manuscript should more explicitly discuss the practical consequences of this modeling decision. Real classroom interactions are rarely free from these influences, and therefore the proposed thresholds should not be interpreted as direct indicators of problematic collaboration. Rather, they represent deviations from an idealized structural baseline. Clarifying the conditions under which these benchmarks are likely to remain informative, and those under which they may become less applicable, would improve the interpretation of the findings.*

Response: We have made the practical consequences of the idealized baseline explicit in Section 3.3. The baseline is most informative when all three members have broadly comparable opportunities to contribute. Assigned roles, tutoring, extended explanations, and differences in task responsibility can instead create expected asymmetries. Accordingly, departure from the reference distribution indicates deviation from the modeled structural baseline but does not by itself establish problematic collaboration.

The revised text also distinguishes the simulator's assumptions from the empirical parameter estimates. Omitting individual and relational differences from the simulator does not establish that such influences are absent from the data used for calibration. Section 6 further explains that the baseline is less directly applicable when task demands or interaction structures intentionally create asymmetry, and that an observed departure cannot identify whether the pattern arises from status, expertise, friendship, personality, social anxiety, or another unmodeled influence. Educational interpretation therefore requires evidence about the particular interaction and its context.

Changes in Manuscript: 1. Section 3.3, p. 10, lines 385–389 "The resulting baseline is most informative when the task offers broadly comparable opportunities for all three members to contribute. Assigned roles, tutoring, extended explanations, or differences in task responsibility can produce expected participation asymmetries, so departure from this baseline does not by itself indicate problematic collaboration."

2. Section 3.3, p. 10, lines 389–391 "Moreover, excluding individual and relational differences from the simulator does not establish that the empirical transition probabilities used to calibrate the model are free from such influences."

3. Section 6, p. 24, lines 929–933 "The baseline is less directly applicable when roles or task demands intentionally create asymmetry, and departure from the reference distribution cannot identify whether the observed pattern arises from exclusion, expertise, personality, friendship, social anxiety, or another unmodeled influence."

Reviewer Comment 4:

Another important limitation concerns the absence of external validation. The manuscript demonstrates that the simulation is internally stable through sensitivity analyses, which is commendable. However, no independent dataset is used to evaluate whether groups exceeding the proposed thresholds actually display poorer collaboration, reduced learning outcomes, or lower-quality interactions. Consequently, the study establishes statistical reference values rather than educationally validated intervention thresholds. This distinction deserves greater emphasis throughout both the Results and Discussion sections. Future work should examine whether groups identified by these benchmark values also exhibit meaningful differences in collaboration quality, learning achievement, or teacher evaluations.

Response: We agree that internal model stability must be distinguished from independent educational validation. We have strengthened this distinction throughout the revised manuscript. Section 4.4 now describes the empirical comparisons as within-sample triangulation, Section 4.6 identifies the parameter-recovery and sensitivity analyses as internal model checks, and Section 6 explicitly acknowledges the absence of an independent classroom dataset linking threshold exceedance to collaboration quality, learning outcomes, or intervention benefit. Accordingly, we now characterize the results as statistical reference values rather than educationally validated intervention thresholds.

We retain the teacher-informed comparison and discourse cases introduced in the first-round revision while clarifying their evidential limits. The teacher-informed benchmark is itself defined using participation-SD cutpoints, and the discourse cases are drawn from the same empirical dataset. Their agreement therefore provides within-sample triangulation rather than validation against an independent educational standard. The revised Limitations section further identifies independent expert coding, teacher judgments, student reports, learning outcomes, and independent classroom datasets as evidence needed to evaluate the educational meaning and external validity of the reference values. No independent external validation is claimed in the present study.

Changes in Manuscript: 1. Section 4.4, p. 15, lines 571–576 "These analyses provide within-sample empirical triangulation rather than independent external validation. Because the teacher-informed benchmark is also defined using cutpoints on participation-share SD, agreement with the simulation criterion should be interpreted as convergence between two operationalizations. The selected discourse cases provide qualitative illustrations of the interaction patterns associated with threshold exceedance."

2. Section 4.4.1, p. 16, lines 624–625 "These are descriptive agreement statistics rather than estimates of diagnostic accuracy against an independent educational standard."

3. Section 6, p. 25, lines 950–951 "No independent classroom dataset in this study links threshold exceedance to poorer collaboration, reduced learning, or the benefit of an intervention."

4. Section 6, p. 25, lines 965–970 (already in first round revision) "Future research could further validate these quantitative evaluation criteria using independent samples and additional educational evidence, such as expert coding of collaboration quality, teacher judgments, student reports of participation experience, and learning outcome measures. Such work could examine whether episodes flagged by the simulation-based criteria are consistently associated with lower perceived participation access, poorer collaborative quality, or weaker learning gains."

Reviewer Comment 5:

Similarly, the interpretation of the proposed 95th percentile thresholds would benefit from additional justification. Although the statistical reasoning appears reasonable, the educational rationale remains somewhat unclear. Readers may reasonably ask why the 95th

percentile rather than the 90th or 97.5th percentile should represent the point at which intervention becomes appropriate. The manuscript should clarify that these thresholds are intended as probabilistic warning indicators rather than definitive diagnostic cutoffs and discuss how different percentile choices might influence practical decision-making.

Response: We have clarified in Section 4.3 that the 95th percentile is a conventional statistical reference conditional on the model, estimated parameters, metric, and discussion length. It is intended as a probabilistic warning indicator rather than a definitive diagnostic or intervention cutoff. It has not been established as an educationally optimal threshold, and exceedance does not imply a 95% probability that a group is inequitable or requires intervention.

The added explanation compares the 90th, 95th, and 97.5th percentiles through their nominal upper-tail probabilities of 10%, 5%, and 2.5%. A lower percentile provides a less conservative screening rule and flags more episodes, whereas a higher percentile provides a more conservative rule and flags fewer. Actual exceedance frequencies may differ from these nominal probabilities because the simulated measures are discrete and the quantiles are estimated. We further explain that practical selection of a warning level requires independent educational evidence and consideration of the consequences of flagging more or fewer episodes. The Discussion's worked example carries the same interpretive qualification. This is a conceptual comparison of alternative warning levels; we have not added an empirical reclassification analysis at the alternative percentiles.

Changes in Manuscript: 1. Section 4.3, p. 14, lines 556–561 "The 95th percentile is used as a conventional upper-tail reference point under the specified model, estimated parameters, metric, and turn count, rather than as an educationally validated intervention cutoff. The 90th, 95th, and 97.5th percentiles correspond to nominal upper-tail probabilities of 10%, 5%, and 2.5% under the reference distribution; exact exceedance frequencies may differ because the simulated measures are discrete and the quantiles are estimated."

2. Section 4.3, p. 14, lines 561–567 "Lower percentile thresholds would flag more episodes, whereas higher thresholds would flag fewer. Selecting an operational warning level therefore requires independent educational evidence and consideration of the consequences of different threshold choices. An observed value above the 95th-percentile reference indicates that it is unusually high relative to the simulated baseline; it does not establish a 95% probability that the group is inequitable or, by itself, justify intervention."

3. Section 5.1, p. 22, lines 811–812 "Such exceedance indicates departure from the specified statistical reference and does not by itself establish poor collaboration or a need for intervention."

Reviewer Comment 6:

I also encourage the authors to further emphasize the distinction between participation equality and collaboration quality. The manuscript appropriately acknowledges that equal participation does not necessarily imply productive collaboration, yet this important point could be discussed more prominently. Groups may exhibit highly balanced turn-taking while simultaneously engaging in superficial or unproductive discussions, whereas high-quality collaborative reasoning may occasionally involve temporary participation asymmetries. Consequently, the proposed benchmark values should be interpreted as indicators of structural participation patterns rather than direct measures of educational effectiveness.

Response: Thank you for this important comment. We have strengthened the distinction between a balanced distribution and productive collaboration in the interpretation of the discourse cases and

in the Discussion. Section 4.4.2 explains that an episode below both thresholds may still contain collaboration-quality problems, while temporary asymmetry may be compatible with productive reasoning when one student explains and others listen, question or build on the explanation.

Section 5.1 adds the corresponding examples: balanced turn counts may accompany superficial exchanges, whereas useful explanations or temporary divisions of roles can produce unequal counts. The indices describe the distribution of turns and addressed opportunities; they do not directly measure reasoning, learning or the educational value of contributions. These revisions clarify the interpretation of the existing four cases without treating either equality or threshold exceedance as evidence of educational effectiveness.

Changes in Manuscript: 1. Section 4.4.2, p. 19, lines 704–711 "An episode may not be flagged for participation or opportunity inequality while still containing other collaboration-quality problems. The criteria should therefore be interpreted as indicators of distributional imbalance, not as comprehensive measures of participation equity, discourse quality, or educational fairness. Conversely, temporary asymmetry may be compatible with productive collaborative reasoning, for example when one student explains an idea and others listen, question, or build on it. Thus, neither a balanced distribution nor threshold exceedance by itself establishes the educational effectiveness of the collaboration."

2. Section 5.1, p. 23, lines 852–856 "Balanced turn counts can accompany superficial exchanges, while a useful extended explanation or temporary division of roles can produce unequal counts within productive collaboration. The indices therefore describe structural participation patterns rather than reasoning quality, learning achievement, or the educational value of contributions."

Reviewer Comment 7:

> *From a methodological perspective, the procedures are generally rigorous and well documented. The estimation of the local transition probabilities appears appropriate, and the sensitivity analyses provide useful evidence regarding model stability. Nevertheless, I would appreciate further discussion regarding the exclusion of students contributing fewer than ten turns during parameter estimation. While the rationale is understandable, these minimally participating students may actually represent precisely the type of participation inequality that the proposed framework ultimately seeks to identify. The potential influence of this exclusion criterion on the estimated transition probabilities should therefore be acknowledged more explicitly.*

Response: We agree that minimally participating students are directly relevant to the inequalities motivating this study. Section 4.2 now specifies the exclusion unit: all participant-level rate estimates from a discussion were excluded from parameter estimation if any member contributed fewer than 10 speaking turns. The cutoff is described as a pragmatic minimum for stabilizing frequency-based estimates rather than a criterion for participation quality. With few eligible events, a single event can substantially change an estimated probability, particularly for the addressing probabilities when speaking turns are scarce.

The revised text explains that pooling the eligible rates was intended to reduce dependence on any single task or group composition, but does not remove individual or relational influences. It also clarifies that the exclusion applied only to parameter estimation: all 245 valid episodes were retained for the empirical assessment. Section 6 acknowledges that this eligibility rule may make the calibration sample less representative of highly asymmetric interactions and identifies sensitivity to alternative eligibility rules as an important direction for future work.

Because we did not re-estimate the parameters under alternative inclusion rules, we do not quantify the direction or magnitude of the effect of this eligibility rule on the estimated transition probabilities. The existing parameter-perturbation analyses examine sensitivity to changes in the parameter values themselves and do not test sensitivity to the exclusion criterion. We therefore present the estimated parameters as calibration values for the stated baseline and explicitly retain this limitation.

Changes in Manuscript: 1. Section 4.2, p. 12, lines 461–464 "When estimating the three parameters, we excluded all participant-level rate estimates from a group discussion if any member contributed fewer than 10 speaking turns. The 10-turn cutoff was a pragmatic minimum intended to improve the stability of frequency-based parameter estimates, rather than a criterion for participation quality."

2. Section 4.2, p. 12, lines 469–472 "Pooling these rates was intended to reduce dependence on any single task or group composition; it does not imply that individual or relational influences were eliminated from the empirical estimates."

3. Section 4.2, p. 12, lines 472–476 "The exclusion applied only to parameter estimation; all 245 valid episodes were retained for the empirical assessment. Because the cutoff conditions parameter estimation on discussions with at least minimal participation from all three members, the resulting estimates should be interpreted as calibration values for the baseline model rather than as estimates characterizing all observed interaction patterns."

4. Section 6, p. 24, lines 920–924 "Within the present sample, parameter estimation included only discussions in which all three members contributed at least 10 speaking turns. This may limit the representativeness of the calibration sample for highly asymmetric interactions. Future work can examine the sensitivity of the parameter estimates and resulting reference distributions to alternative eligibility rules."

Reviewer Comment 8:

> *The interpretation of the findings is generally careful and avoids unwarranted causal claims. However, some conclusions occasionally extend beyond what the evidence can directly support. The manuscript sometimes implies that groups exceeding the benchmark values are experiencing participation inequality requiring intervention. Strictly speaking, the study demonstrates only that these groups exhibit greater inequality than would be expected under the simulated baseline conditions. Whether such deviations correspond to educationally meaningful problems remains an empirical question that future research must address. Moderating this language would improve the overall rigor of the manuscript.*

Response: We have moderated the practical interpretation of the reference values. The threshold explanation distinguishes a statistical warning indicator from evidence that intervention is needed. The Discussion presents monitoring and scaffolding as possible applications that require independent evaluation.

In particular, Section 5.1 no longer prescribes a 30–50-turn monitoring window: it states that the findings do not establish an effective window length and that the value of proposed monitoring or support mechanisms remains to be tested. It also specifies that a useful basis for closer examination does not establish when or how support should be provided. Section 5.2 explains that the proportion of flagged episodes is an exceedance frequency relative to the specified reference, rather than the proportion requiring or benefiting from scaffolding. Its concluding statement limits the contribution

to a context-specific statistical basis for examining participation dynamics and identifies educational evaluation as future work.

Response: We have moderated the interpretation of the reference values throughout the manuscript. The revised threshold explanation now distinguishes exceedance of the simulated baseline from evidence of an educationally meaningful problem or a need for intervention. Monitoring and scaffolding are presented only as possible applications that require independent evaluation.

In addition, in Section 5.1, we no longer prescribe a 30–50-turn monitoring window. Instead, we state that the present findings do not establish an effective monitoring-window length and that the usefulness of monitoring or support mechanisms remains to be tested. We also clarify that the reference values may support closer examination of participation patterns but do not determine when or how support should be provided. Section 5.2 further explains that the proportion of flagged episodes represents exceedance of the specified statistical reference, not the proportion of groups requiring or benefiting from scaffolding. The section now concludes by limiting the contribution to a context-specific statistical basis for examining participation dynamics, with the educational meaning of departures from that basis left for future validation.

Changes in Manuscript: 1. Section 5.1, p. 22, lines 828–831 "The observed early fluctuations suggest a possible direction for research on real-time monitoring, but this study does not establish an effective monitoring-window length. Whether such monitoring helps educators identify meaningful concerns or provide useful support remains to be tested independently."

2. Section 5.1, p. 23, lines 846–848 "These reference values may help organize closer examination of participation patterns, but their usefulness for determining the timing or effectiveness of scaffolding has not been established."

3. Section 5.2, p. 23, lines 866–873 "When the simulation-based reference criterion was applied to the empirical data, participation inequality was flagged more frequently than opportunity inequality overall. These frequencies describe exceedance of the respective simulation-based reference distributions and should not be interpreted as direct evidence of poor collaboration or learning. Whether a flagged episode represents educationally problematic participation requires evidence beyond turn-count distributions. Social awareness tools and communication strategies are possible forms of support [1], but the present analysis does not establish that flagged groups require such support or would benefit from it."

4. Section 5.2, p. 24, lines 885–889 "These findings provide a context-specific statistical basis for examining participation dynamics, while the educational meaning of departures from that basis remains an empirical question. Future research can evaluate whether reference values generated by this procedure support equitable and productive interaction using independent measures of collaboration quality, student experience, and learning."

Reviewer Comment 9:

> *Finally, while the limitations section has improved, it would benefit from a more comprehensive discussion of the study's boundaries. In particular, the authors should explicitly acknowledge the cultural specificity of the sample, the restriction to triadic groups, the exclusive reliance on turn-based indicators rather than qualitative discourse features, the dependence of the simulation on estimated transition probabilities, and the absence of external validation using independent classroom datasets.*

Response: We have brought the requested study boundaries together in Section 6. The revised Limitations section explicitly identifies the educational and cultural specificity of the calibration

sample; the restriction to triadic groups; the reliance on turn counts and addressed opportunities, which do not capture the content, timing, or interactional function of contributions; the dependence of the simulated reference distributions on empirically estimated transition probabilities; and the absence of external validation using independent classroom datasets.

The section also acknowledges that the fewer-than-ten-turn eligibility rule may limit the representativeness of the calibration sample and that assigned roles or task demands can create expected asymmetries. We distinguish the internal parameter-recovery and sensitivity analyses from evidence of generalizability or educational validity. Future work therefore includes recalibration in other populations and interaction settings, examination of alternative eligibility rules, and validation with independent classroom data and evidence concerning collaboration quality, students' participation experiences, and learning outcomes.

Changes in Manuscript: 1. Section 6, p. 24, lines 906–917 "Second, the empirical parameter estimates and benchmark values used in this study were derived from 117 fourth-grade students in two mainland-China classrooms, working in heterogeneous triads on mathematics and reading tasks. Although the stability analysis suggests that the proposed benchmarks are not highly sensitive to reasonable variations in the estimated parameters, this internal robustness does not establish broad external generalizability. Future research should validate and, where necessary, recalibrate both the parameter estimates and the resulting benchmarks across different age groups, cultural contexts, task types, and instructional settings before applying them to broader collaborative learning contexts. In addition, the study exclusively focuses on triadic groups, while group size is a pivotal factor influencing participation equity. Future research endeavors should aim to establish quantitative evaluation criteria for other common small group sizes, such as four, five, and six-person groups."

2. Section 6, p. 24, lines 920–924 "Within the present sample, parameter estimation included only discussions in which all three members contributed at least 10 speaking turns. This may limit the representativeness of the calibration sample for highly asymmetric interactions. Future work can examine the sensitivity of the parameter estimates and resulting reference distributions to alternative eligibility rules."

3. Section 6, p. 25, lines 950–956 "No independent classroom dataset in this study links threshold exceedance to poorer collaboration, reduced learning, or the benefit of an intervention. Furthermore, the confidence of these recommended thresholds is based on simulated results, which rely on model assumptions and may not capture all real-world variations in turn-taking behavior. The reference values also depend on estimated transition probabilities and the chosen percentile; internal sensitivity checks do not establish robustness to changes in population, eligibility rules, or educational use."

4. Section 6, p. 25, lines 956–962 (already in first round revision) "Moreover, the proposed benchmarks are based on the structural distribution of speaking turns and addressed opportunities, rather than on the qualitative content, timing, or interactional function of participants' contributions. The present coding does not distinguish, for example, whether a turn involved elaboration, questioning, repair, interruption, or a brief acknowledgment. Nor does it capture whether silence reflected disengagement, exclusion, listening, or productive reflection."

Conclusion

We again thank you for your insightful comments and constructive suggestions.
