# Supplementary material for: How unequal is unequal? Turn-taking simulation for benchmarking participation and opportunity inequality in small-group collaboration
Source: PLoS One. 2026 Oct 23;21(10):e0361205. doi: 10.1371/journal.pone.0361205 (PMC13693943; doi:10.1371/journal.pone.0361205)
Supplement: Supplementary file 3 [file pone.0361205.s001.docx]

The manuscript requires major revision before it can be considered for publication in an SSCI-indexed journal. The primary concerns relate to limited empirical validation, insufficient statistical rigor, ecological validity of the model, and non-compliance with data availability requirements.

1 Limited Empirical Validation

The most critical limitation is the weak link between simulation outputs and real-world data:

- The empirical dataset is used mainly for parameter estimation, not for validating the proposed thresholds.
- There is no inferential testing demonstrating that the thresholds meaningfully detect inequality in authentic classroom settings.
- The claim that ~66% of groups show inequality is descriptive and lacks criterion validation.

*Strengthen empirical validation by:

- Testing whether thresholds predict observable outcomes (e.g., learning gains, engagement)
- Comparing flagged vs. non-flagged groups using inferential statistics
- Including qualitative validation (e.g., discourse analysis)

2 Ecological Validity of the Model

The simulation assumes “ideal equity” by excluding Individual characteristics (e.g., ability, personality) and Social dynamics (e.g., status, friendship)

While methodologically convenient, this assumption oversimplifies real classroom interactions and limits applicability of the derived benchmarks

*You need to provide stronger justification for excluding these variables, clearly delimit the scope of conclusions and consider incorporating variability in future modeling

3 Statistical Rigor and Analytical Depth

The statistical analysis is technically correct but insufficiently rigorous:

- Over-reliance on descriptive statistics and simulation percentiles
- No inferential modeling (e.g., regression, multilevel analysis)
- No effect sizes
- No comparison with alternative inequality metrics (e.g., Gini coefficient)

*You should introduce inferential statistical analyses, justify metric choice more robustly and compare results across multiple inequality indices

4 Data Availability and Transparency

The manuscript states that data are not publicly available. Provide a publicly accessible, de-identified dataset, or offer a clear, policy-compliant justification with institutional restrictions

5 Generalizability

The study is limited to fourth-grade students, a single cultural context (China) and specific task types. Yet, the discussion occasionally implies broader applicability.

*You should use moderate claims, clearly define boundary conditions and expand discussion of cultural/task variability

6 Writing and Style is generally clear, but some sections are verbose and repetitive. Methodology could be streamlined for readability and there are some minor grammatical inconsistencies

7 Literature Review could engage more critically with competing metrics. You should include more recent (2023–2025) SSCI studies

8 Ensure consistent use of “inequality” vs. “inequity”, “participation” vs. “contribution”

9 Figures are useful but require clearer captions and more explicit interpretation in the text
