# Supplementary material for: How unequal is unequal? Turn-taking simulation for benchmarking participation and opportunity inequality in small-group collaboration
Source: PLoS One. 2026 Oct 23;21(10):e0361205. doi: 10.1371/journal.pone.0361205 (PMC13693943; doi:10.1371/journal.pone.0361205)
Supplement: Supplementary file 4 [file pone.0361205.s003.docx]

I appreciate the considerable effort the authors have invested in revising the manuscript. Compared with the previous version, the manuscript has improved substantially in terms of its conceptual organization, methodological transparency, and theoretical justification. The revised literature review more clearly distinguishes participation equality from opportunity equality, the rationale for the simulation framework has been strengthened, and the methodological procedures are described in greater detail. The inclusion of additional explanations regarding the local turn-taking parameters and the expanded discussion of the simulation model considerably enhance the readability of the manuscript. Despite these strengths, several important issues remain before the manuscript is suitable for publication.

*The most significant concern relates to the generalizability of the proposed benchmark values. The simulation parameters are estimated from a relatively specific empirical context consisting of 117 fourth-grade students from only two classrooms in mainland China who worked in heterogeneous triads on mathematics and reading tasks. Nevertheless, throughout the manuscript the proposed benchmark values are presented in a manner that could easily be interpreted as generally applicable reference thresholds. Since conversational norms, classroom culture, instructional practices, age, task characteristics, and cultural background are all likely to influence the estimated transition probabilities, the proposed thresholds should be presented more cautiously. Rather than describing them as benchmarks for participation inequality in general, they should be explicitly characterized as preliminary reference values derived from one educational context. The discussion would benefit from a more explicit consideration of how these benchmark values may vary across educational settings and how future studies might recalibrate the model for different populations and collaborative environments.

*Although the authors have expanded the methodological justification for selecting the standard deviation as the primary inequality measure, I remain unconvinced that the manuscript sufficiently explains why this metric should be preferred over alternative measures that are already well established in the literature, such as the Gini coefficient, entropy-based indices, or the coefficient of variation. The literature review appropriately acknowledges these alternatives; however, the manuscript ultimately adopts the standard deviation without fully demonstrating why it is particularly advantageous for simulation-based benchmarking. A more explicit discussion of its statistical properties, interpretability, computational simplicity, and suitability for educational applications would strengthen the methodological argument. Even a brief supplementary comparison illustrating that similar benchmark patterns emerge when alternative inequality indices are used would substantially reinforce confidence in the proposed approach.

*The simulation framework itself is elegant but necessarily simplified. The authors correctly explain that the purpose is to establish an idealized baseline by excluding individual characteristics such as academic status, personality, friendship, social anxiety, and gender. While this simplification is understandable, the manuscript should more explicitly discuss the practical consequences of this modeling decision. Real classroom interactions are rarely free from these influences, and therefore the proposed thresholds should not be interpreted as direct indicators of problematic collaboration. Rather, they represent deviations from an idealized structural baseline. Clarifying the conditions under which these benchmarks are likely to remain informative, and those under which they may become less applicable, would improve the interpretation of the findings.

*Another important limitation concerns the absence of external validation. The manuscript demonstrates that the simulation is internally stable through sensitivity analyses, which is commendable. However, no independent dataset is used to evaluate whether groups exceeding the proposed thresholds actually display poorer collaboration, reduced learning outcomes, or lower-quality interactions. Consequently, the study establishes statistical reference values rather than educationally validated intervention thresholds. This distinction deserves greater emphasis throughout both the Results and Discussion sections. Future work should examine whether groups identified by these benchmark values also exhibit meaningful differences in collaboration quality, learning achievement, or teacher evaluations.

*Similarly, the interpretation of the proposed 95th percentile thresholds would benefit from additional justification. Although the statistical reasoning appears reasonable, the educational rationale remains somewhat unclear. Readers may reasonably ask why the 95th percentile rather than the 90th or 97.5th percentile should represent the point at which intervention becomes appropriate. The manuscript should clarify that these thresholds are intended as probabilistic warning indicators rather than definitive diagnostic cutoffs and discuss how different percentile choices might influence practical decision-making.

*I also encourage the authors to further emphasize the distinction between participation equality and collaboration quality. The manuscript appropriately acknowledges that equal participation does not necessarily imply productive collaboration, yet this important point could be discussed more prominently. Groups may exhibit highly balanced turn-taking while simultaneously engaging in superficial or unproductive discussions, whereas high-quality collaborative reasoning may occasionally involve temporary participation asymmetries. Consequently, the proposed benchmark values should be interpreted as indicators of structural participation patterns rather than direct measures of educational effectiveness.

*From a methodological perspective, the procedures are generally rigorous and well documented. The estimation of the local transition probabilities appears appropriate, and the sensitivity analyses provide useful evidence regarding model stability. Nevertheless, I would appreciate further discussion regarding the exclusion of students contributing fewer than ten turns during parameter estimation. While the rationale is understandable, these minimally participating students may actually represent precisely the type of participation inequality that the proposed framework ultimately seeks to identify. The potential influence of this exclusion criterion on the estimated transition probabilities should therefore be acknowledged more explicitly.

*The interpretation of the findings is generally careful and avoids unwarranted causal claims. However, some conclusions occasionally extend beyond what the evidence can directly support. The manuscript sometimes implies that groups exceeding the benchmark values are experiencing participation inequality requiring intervention. Strictly speaking, the study demonstrates only that these groups exhibit greater inequality than would be expected under the simulated baseline conditions. Whether such deviations correspond to educationally meaningful problems remains an empirical question that future research must address. Moderating this language would improve the overall rigor of the manuscript.

Finally, while the limitations section has improved, it would benefit from a more comprehensive discussion of the study's boundaries. In particular, the authors should explicitly acknowledge the cultural specificity of the sample, the restriction to triadic groups, the exclusive reliance on turn-based indicators rather than qualitative discourse features, the dependence of the simulation on estimated transition probabilities, and the absence of external validation using independent classroom datasets.

Overall, I believe this revised manuscript represents a significant improvement over the previous submission and makes an original contribution to the literature on collaborative learning and learning analytics. The methodological innovation is noteworthy, and the proposed simulation framework has considerable potential to support future research and educational practice. Nevertheless, several conceptual and interpretative issues remain unresolved, particularly regarding the generalizability and educational interpretation of the proposed benchmark values. Addressing these concerns would substantially strengthen the manuscript and increase its impact within the field.
