# Supplementary material for: How unequal is unequal? Turn-taking simulation for benchmarking participation and opportunity inequality in small-group collaboration
Source: PLoS One. 2026 Oct 23;21(10):e0361205. doi: 10.1371/journal.pone.0361205 (PMC13693943; doi:10.1371/journal.pone.0361205)
Supplement: Peer review history

# Peer review: Decision Letter 0

**DOI:** 10.1371/journal.pone.0361205.r001

**Authors:** Kristiawan Indriyanto

11 May 2026

Dear Dr. Hu,

Thank you for submitting your manuscript to PLOS ONE. After careful consideration, we feel that it has merit but does not fully meet PLOS ONE’s publication criteria as it currently stands. Therefore, we invite you to submit a revised version of the manuscript that addresses the points raised during the review process.

==============================

**Please follow the reviewers' comments and suggestions to improve the quality of the manuscript.**

==============================

Please submit your revised manuscript by Jun 25 2026 11:59PM. If you will need more time than this to complete your revisions, please reply to this message or contact the journal office at plosone@plos.org. When you're ready to submit your revision, log on to https://www.editorialmanager.com/pone/ and select the 'Submissions Needing Revision' folder to locate your manuscript file.

- A letter that responds to each point raised by the academic editor and reviewer(s). You should upload this letter as a separate file labeled 'Response to Reviewers'.
- A marked-up copy of your manuscript that highlights changes made to the original version. You should upload this as a separate file labeled 'Revised Manuscript with Track Changes'.
- An unmarked version of your revised paper without tracked changes. You should upload this as a separate file labeled 'Manuscript'.

If you would like to make changes to your financial disclosure, please include your updated statement in your cover letter. Guidelines for resubmitting your figure files are available below the reviewer comments at the end of this letter.

If applicable, we recommend that you deposit your laboratory protocols in protocols.io to enhance the reproducibility of your results. Protocols.io assigns your protocol its own identifier (DOI) so that it can be cited independently in the future. For instructions see: https://journals.plos.org/plosone/s/submission-guidelines#loc-laboratory-protocols. Additionally, PLOS ONE offers an option for publishing peer-reviewed Lab Protocol articles, which describe protocols hosted on protocols.io. Read more information on sharing protocols at https://plos.org/protocols?utm_medium=editorial-email&utm_source=authorletters&utm_campaign=protocols.

As the corresponding author, your ORCID iD is verified in the submission system and will appear in the published article. PLOS supports the use of ORCID, and we encourage all coauthors to register for an ORCID iD and use it as well. Please encourage your coauthors to verify their ORCID iD within the submission system before final acceptance, as unverified ORCID iDs will not appear in the published article. *Only*  the individual author can complete the verification step; PLOS staff *cannot*  verify ORCID iDs on behalf of authors.

We look forward to receiving your revised manuscript.

Kind regards,

Kristiawan Indriyanto

Academic Editor

PLOS One

Journal Requirements:

When submitting your revision, we need you to address these additional requirements.

1. Please ensure that your manuscript meets PLOS ONE's style requirements, including those for file naming. The PLOS ONE style templates can be found at

https://journals.plos.org/plosone/s/file?id=wjVg/PLOSOne_formatting_sample_main_body.pdf and

https://journals.plos.org/plosone/s/file?id=ba62/PLOSOne_formatting_sample_title_authors_affiliations.pdf

2. Please include a complete copy of PLOS’ questionnaire on inclusivity in global research in your revised manuscript. Our policy for research in this area aims to improve transparency in the reporting of research performed outside of researchers’ own country or community. The policy applies to researchers who have travelled to a different country to conduct research, research with Indigenous populations or their lands, and research on cultural artefacts. The questionnaire can also be requested at the journal’s discretion for any other submissions, even if these conditions are not met.  Please find more information on the policy and a link to download a blank copy of the questionnaire here: https://journals.plos.org/plosone/s/best-practices-in-research-reporting. Please upload a completed version of your questionnaire as Supporting Information when you resubmit your manuscript.

3. Please note that PLOS One has specific guidelines on code sharing for submissions in which author-generated code underpins the findings in the manuscript. In these cases, we expect all author-generated code to be made available without restrictions upon publication of the work. Please review our guidelines at https://journals.plos.org/plosone/s/materials-and-software-sharing#loc-sharing-code and ensure that your code is shared in a way that follows best practice and facilitates reproducibility and reuse.

4. In the online submission form you indicate that your data is not available for proprietary reasons and have provided a contact point for accessing this data. Please note that your current contact point is a co-author on this manuscript. According to our Data Policy, the contact point must not be an author on the manuscript and must be an institutional contact, ideally not an individual. Please revise your data statement to a non-author institutional point of contact, such as a data access or ethics committee, and send this to us via return email. Please also include contact information for the third party organization, and please include the full citation of where the data can be found.

5. If the reviewer comments include a recommendation to cite specific previously published works, please review and evaluate these publications to determine whether they are relevant and should be cited. There is no requirement to cite these works unless the editor has indicated otherwise.

[Note: HTML markup is below. Please do not edit.]

Reviewers' comments:

Reviewer's Responses to Questions

**Comments to the Author**

1. Is the manuscript technically sound, and do the data support the conclusions?

Reviewer #1: Partly

Reviewer #2: Partly

**********

2. Has the statistical analysis been performed appropriately and rigorously?

Reviewer #1: No

Reviewer #2: No

**********

3. Have the authors made all data underlying the findings in their manuscript fully available?

The PLOS Data policy

Reviewer #1: No

Reviewer #2: No

**********

4. Is the manuscript presented in an intelligible fashion and written in standard English?

Reviewer #1: Yes

Reviewer #2: Yes

**********

Reviewer #1: This manuscript addresses an important and underexplored issue in collaborative learning research, namely the lack of quantitative benchmarks for determining participation equality. The integration of empirical classroom data with an agent-based modeling approach is innovative and has the potential to make a meaningful contribution to the learning analytics and CSCL literature. The manuscript is generally well-structured and clearly written, and the research problem is well-motivated. However, several conceptual, methodological, and reporting issues need to be addressed before the manuscript can meet SSCI publication standards.

First, while the modeling framework is technically interesting, the study relies heavily on simulation to derive its main conclusions. The empirical dataset appears to be used primarily for parameter estimation rather than for rigorous validation of the proposed thresholds. This creates a disconnect between the real-world data and the claims made. The manuscript would be significantly strengthened by incorporating stronger empirical validation, for example by testing whether the proposed inequality thresholds predict meaningful educational outcomes or observable interactional imbalances in actual classroom data.

Second, the decision to exclude Type I and Type II individual factors from the simulation in order to construct “ideal equity” conditions is understandable from a modeling perspective, yet it raises concerns about ecological validity. Participation in real classrooms is inherently shaped by individual, social, and cultural variables. The current model may therefore oversimplify the phenomenon it aims to represent. The authors should more explicitly discuss the implications of this abstraction and clearly delimit the scope of their claims.

Third, although the statistical procedures used are generally appropriate, the analytical rigor remains limited for a high-impact journal. The study relies largely on descriptive statistics, normality tests, and simulation percentiles. There is no use of inferential statistical modeling, no reporting of effect sizes, and no comparison with alternative inequality measures such as the Gini coefficient. Providing a stronger statistical justification for the chosen metrics and triangulating results with alternative indices would enhance the robustness of the findings.

Fourth, the issue of data availability is a significant concern. The manuscript states that the data cannot be publicly shared and are only available upon request. This does not align with PLOS data transparency requirements. The authors need to either provide a publicly accessible, de-identified dataset or offer a much clearer and policy-compliant justification for the restriction.

Fifth, the generalizability of the findings is limited. The sample consists of fourth-grade students from a specific educational context, and the tasks are relatively narrow in scope. While this is acknowledged in the limitations section, the discussion tends to present the proposed benchmarks as broadly applicable. The authors should moderate their claims and more carefully contextualize the applicability of their model.

Finally, minor revisions are needed in language and style. While the manuscript is generally clear, some sections, particularly the methodology and literature review, could be streamlined to improve readability. There are occasional redundancies and minor grammatical inconsistencies that should be corrected.

In conclusion, the manuscript presents a novel and potentially impactful idea, but it requires substantial revision in terms of empirical grounding, statistical rigor, and data transparency before it can be considered for publication.

Reviewer #2: Thank you for the opportunity to review the manuscript entitled “Developing Quantitative Equality Criteria in Small-Group Collaboration: A Turn-Taking Simulation Approach.” This study addresses an important and timely issue in collaborative learning research: how to quantitatively evaluate participation equity in small-group interaction. The manuscript proposes a simulation-based framework for establishing threshold values for participation inequality and opportunity inequality in triadic group discussions using agent-based modeling and turn-taking simulations.

Overall, I believe this manuscript has a meaningful and potentially valuable contribution. In particular, the paper attempts to operationalize participation equity into measurable quantitative criteria, which could have important implications for learning analytics, group awareness tools, and teacher orchestration systems. The paper is also clearly organized, and the computational modeling process is generally easy to follow.

However, despite these strengths, I believe the manuscript currently has several major conceptual and methodological issues that substantially weaken the validity of its conclusions. In particular, the theoretical assumptions underlying the turn-taking model require much stronger justification, and the relationship between the simulation model and real educational equity remains insufficiently validated. Therefore, I recommend a major revision.

Major Concerns

1. The theoretical justification for the three dialogue parameters is insufficient.

The entire simulation framework relies heavily on three parameters: Prec, Ptb, and Ptg. The manuscript argues that these parameters represent “features intrinsic to human dialogue” (p. 7). However, the theoretical basis for selecting only these three interaction mechanisms is not sufficiently justified.

Three-person collaborative interaction is substantially more complex than the proposed model suggests. Real collaborative dialogue includes many additional interactional processes, such as interruption, coalition formation, dominance accumulation, repair, epistemic positioning, silence, emotional reciprocity, and long-term interactional dependencies. The current model reduces interaction dynamics primarily to immediate turn-transition probabilities.

While simplification is understandable in simulation research, the manuscript needs to more clearly explain why these three parameters are theoretically sufficient to represent equitable turn-taking dynamics. At present, the selection of these parameters appears somewhat arbitrary and under-theorized.

I strongly recommend that the authors provide a more substantial grounding in conversation analysis, dialogic interaction theory, or turn-taking literature to justify the modeling assumptions.

2. The manuscript makes overly strong claims about “intrinsic dialogue features.”

A major conceptual issue is that the three parameters are estimated from real classroom discussions that already contain participation inequalities, social dynamics, status effects, and interpersonal asymmetries. However, the manuscript later treats these parameters as representing idealized “intrinsic” properties of human dialogue and uses them to simulate “ideally equitable” group interaction.

This creates an important conceptual tension.

The empirical classroom data used to estimate Prec, Ptb, and Ptg likely already reflect the influence of academic status, friendship, confidence, anxiety, dominance, and other social factors. Averaging across groups does not necessarily remove these structural inequalities. Therefore, it is difficult to conclude that the resulting parameters represent purely dialogue-intrinsic mechanisms.

The manuscript would benefit from a more cautious interpretation. Instead of framing these parameters as universal intrinsic dialogue features, the authors may consider describing them as simplified approximations of turn-taking tendencies observed in their dataset.

3. The interaction model is overly simplified and ignores longer-term interaction dynamics.

The proposed simulation model effectively functions as a first-order Markov process, where the next conversational action depends primarily on the immediately preceding turn. However, collaborative interaction is often shaped by cumulative history and emergent group dynamics.

For example, many interactional phenomena relevant to participation equity emerge over time, including:

* dominance stabilization,

* coalition formation,

* role differentiation,

* increasing withdrawal,

* evolving participation norms,

* interactional repair processes.

Ironically, the manuscript itself discusses “lock-in” effects in participation dynamics later in the discussion section, yet the simulation model does not meaningfully incorporate history-dependent mechanisms.

The authors should acknowledge more explicitly that the current model captures only a highly simplified local structure of turn-taking rather than the broader temporal dynamics of collaboration.

4. The operationalization of “equity” is too narrow.

The manuscript operationalizes participation equity primarily through the standard deviation of speaking-turn distributions. While this provides a convenient quantitative metric, it risks oversimplifying the educational meaning of equity.

The literature on collaborative learning has long recognized that equal speaking frequency does not necessarily imply equitable participation. Students may contribute differently through:

* active listening,

* reflection,

* coordination,

* summarization,

* questioning,

* epistemic mediation,

* or productive silence.

The manuscript briefly acknowledges this issue in the literature review, but the later modeling framework still equates equity largely with quantitative balance in turn counts.

This limitation should be discussed much more explicitly. Otherwise, the manuscript risks implying that equitable collaboration can be sufficiently reduced to distributional equality of speaking turns.

5. The proposed thresholds lack educational validation.

The manuscript derives participation inequality thresholds from simulation percentiles and proposes that groups exceeding the 95th percentile threshold can be considered inequitable with “95% confidence.”

However, these thresholds are statistically simulated rather than educationally validated.

At present, the manuscript does not demonstrate whether the proposed thresholds meaningfully correspond to:

* teacher judgments of inequity,

* expert coding of collaboration quality,

* student experiences,

* or learning outcomes.

As a result, the current thresholds should be interpreted more cautiously as simulation-based reference distributions rather than validated educational criteria.

I strongly encourage the authors to discuss this limitation more carefully and avoid overstating the practical interpretability of the thresholds.

6. The manuscript would benefit from stronger qualitative or empirical triangulation.

Relatedly, the paper currently relies almost entirely on computational simulation and descriptive statistics. Additional validation approaches would substantially strengthen the contribution.

For example, the authors could:

* compare simulated inequity classifications with teacher evaluations,

* analyze whether identified inequitable groups show poorer collaboration quality,

* or conduct qualitative analyses of groups near versus above threshold values.

Without such triangulation, the educational significance of the simulation outputs remains somewhat uncertain.

In summary, I believe this manuscript addresses an important problem and proposes an interesting computational approach for operationalizing participation equity. However, the current version relies on several strong theoretical assumptions that are insufficiently justified, and the educational interpretation of the proposed thresholds remains underdeveloped. I therefore recommend major revision before the manuscript can be considered for publication.

**********

what does this mean?). If published, this will include your full peer review and any attached files.

If you choose “no”, your identity will remain anonymous but your review may still be made public.

**Do you want your identity to be public for this peer review?**  For information about this choice, including consent withdrawal, please see our Privacy Policy

Reviewer #1: No

Reviewer #2: No

**********

[NOTE: If reviewer comments were submitted as an attachment file, they will be attached to this email and accessible via the submission site. Please log into your account, locate the manuscript record, and check for the action link "View Attachments". If this link does not appear, there are no attachment files.]

To ensure your figures meet our technical requirements, please review our figure guidelines: https://journals.plos.org/plosone/s/figures

You may also use PLOS’s free figure tool, NAAS, to help you prepare publication quality figures: https://journals.plos.org/plosone/s/figures#loc-tools-for-figure-preparation.

NAAS will assess whether your figures meet our technical requirements by comparing each figure against our figure specifications.

Attachment Submitted filename: report.docx

# Author comment: Author response to Decision Letter 1

**DOI:** 10.1371/journal.pone.0361205.r002

28 Jun 2026

Please see the attached response letter.

Attachment Submitted filename: RESPONSE LETTER_PLOSONE.pdf

# Peer review: Decision Letter 1

**DOI:** 10.1371/journal.pone.0361205.r003

**Authors:** Kristiawan Indriyanto

7 Sep 2026

PONE-D-26-11209R1

How unequal is unequal? Turn-taking simulation for benchmarking participation and opportunity inequality in small-group collaboration

PLOS One?

Dear Dr. Hu,

Thank you for submitting your manuscript to PLOS One. After careful consideration, we feel that it has merit but does not fully meet PLOS One’s publication criteria as it currently stands. Therefore, we invite you to submit a revised version of the manuscript that addresses the points raised during the review process.

Please revise your paper as suggested by the reviewer.

Please submit your revised manuscript by Oct 22 2026 11:59PM. If you will need more time than this to complete your revisions, please reply to this message or contact the journal office at plosone@plos.org. When you're ready to submit your revision, log on to https://www.editorialmanager.com/pone/ and select the 'Submissions Needing Revision' folder to locate your manuscript file.

- A letter that responds to each point raised by the academic editor and reviewer(s). You should upload this letter as a separate file labeled 'Response to Reviewers'.
- A marked-up copy of your manuscript that highlights changes made to the original version. You should upload this as a separate file labeled 'Revised Manuscript with Track Changes'.
- An unmarked version of your revised paper without tracked changes. You should upload this as a separate file labeled 'Manuscript'.

If you would like to make changes to your financial disclosure, please include your updated statement in your cover letter. Guidelines for resubmitting your figure files are available below the reviewer comments at the end of this letter.

If applicable, we recommend that you deposit your laboratory protocols in protocols.io to enhance the reproducibility of your results. Protocols.io assigns your protocol its own identifier (DOI) so that it can be cited independently in the future. For instructions see: https://journals.plos.org/plosone/s/submission-guidelines#loc-laboratory-protocols. Additionally, PLOS One offers an option for publishing peer-reviewed Lab Protocol articles, which describe protocols hosted on protocols.io. Read more information on sharing protocols at https://plos.org/protocols?utm_medium=editorial-email&utm_source=authorletters&utm_campaign=protocols.

As the corresponding author, your ORCID iD is verified in the submission system and will appear in the published article. PLOS supports the use of ORCID, and we encourage all coauthors to register for an ORCID iD and use it as well. Please encourage your coauthors to verify their ORCID iD within the submission system before final acceptance, as unverified ORCID iDs will not appear in the published article. *Only*  the individual author can complete the verification step; PLOS staff *cannot*  verify ORCID iDs on behalf of authors.

We look forward to receiving your revised manuscript.

Kind regards,

Kristiawan Indriyanto

Academic Editor

PLOS One

Journal Requirements:

If the reviewer comments include a recommendation to cite specific previously published works, please review and evaluate these publications to determine whether they are relevant and should be cited. There is no requirement to cite these works unless the editor has indicated otherwise.

[Note: HTML markup is below. Please do not edit.]

Reviewers' comments:

Reviewer's Responses to Questions

**Comments to the Author**

Reviewer #1: (No Response)

**********

2. Is the manuscript technically sound, and do the data support the conclusions?

Reviewer #1: Partly

**********

3. Has the statistical analysis been performed appropriately and rigorously?

Reviewer #1: No

**********

4. Have the authors made all data underlying the findings in their manuscript fully available?

The PLOS Data policy

Reviewer #1: Yes

**********

5. Is the manuscript presented in an intelligible fashion and written in standard English?

Reviewer #1: Yes

**********

Reviewer #1: I appreciate the considerable effort the authors have invested in revising the manuscript. Compared with the previous version, the manuscript has improved substantially in terms of its conceptual organization, methodological transparency, and theoretical justification. The revised literature review more clearly distinguishes participation equality from opportunity equality, the rationale for the simulation framework has been strengthened, and the methodological procedures are described in greater detail. The inclusion of additional explanations regarding the local turn-taking parameters and the expanded discussion of the simulation model considerably enhance the readability of the manuscript. Despite these strengths, several important issues remain before the manuscript is suitable for publication.

*The most significant concern relates to the generalizability of the proposed benchmark values. The simulation parameters are estimated from a relatively specific empirical context consisting of 117 fourth-grade students from only two classrooms in mainland China who worked in heterogeneous triads on mathematics and reading tasks. Nevertheless, throughout the manuscript the proposed benchmark values are presented in a manner that could easily be interpreted as generally applicable reference thresholds. Since conversational norms, classroom culture, instructional practices, age, task characteristics, and cultural background are all likely to influence the estimated transition probabilities, the proposed thresholds should be presented more cautiously. Rather than describing them as benchmarks for participation inequality in general, they should be explicitly characterized as preliminary reference values derived from one educational context. The discussion would benefit from a more explicit consideration of how these benchmark values may vary across educational settings and how future studies might recalibrate the model for different populations and collaborative environments.

*Although the authors have expanded the methodological justification for selecting the standard deviation as the primary inequality measure, I remain unconvinced that the manuscript sufficiently explains why this metric should be preferred over alternative measures that are already well established in the literature, such as the Gini coefficient, entropy-based indices, or the coefficient of variation. The literature review appropriately acknowledges these alternatives; however, the manuscript ultimately adopts the standard deviation without fully demonstrating why it is particularly advantageous for simulation-based benchmarking. A more explicit discussion of its statistical properties, interpretability, computational simplicity, and suitability for educational applications would strengthen the methodological argument. Even a brief supplementary comparison illustrating that similar benchmark patterns emerge when alternative inequality indices are used would substantially reinforce confidence in the proposed approach.

*The simulation framework itself is elegant but necessarily simplified. The authors correctly explain that the purpose is to establish an idealized baseline by excluding individual characteristics such as academic status, personality, friendship, social anxiety, and gender. While this simplification is understandable, the manuscript should more explicitly discuss the practical consequences of this modeling decision. Real classroom interactions are rarely free from these influences, and therefore the proposed thresholds should not be interpreted as direct indicators of problematic collaboration. Rather, they represent deviations from an idealized structural baseline. Clarifying the conditions under which these benchmarks are likely to remain informative, and those under which they may become less applicable, would improve the interpretation of the findings.

*Another important limitation concerns the absence of external validation. The manuscript demonstrates that the simulation is internally stable through sensitivity analyses, which is commendable. However, no independent dataset is used to evaluate whether groups exceeding the proposed thresholds actually display poorer collaboration, reduced learning outcomes, or lower-quality interactions. Consequently, the study establishes statistical reference values rather than educationally validated intervention thresholds. This distinction deserves greater emphasis throughout both the Results and Discussion sections. Future work should examine whether groups identified by these benchmark values also exhibit meaningful differences in collaboration quality, learning achievement, or teacher evaluations.

*Similarly, the interpretation of the proposed 95th percentile thresholds would benefit from additional justification. Although the statistical reasoning appears reasonable, the educational rationale remains somewhat unclear. Readers may reasonably ask why the 95th percentile rather than the 90th or 97.5th percentile should represent the point at which intervention becomes appropriate. The manuscript should clarify that these thresholds are intended as probabilistic warning indicators rather than definitive diagnostic cutoffs and discuss how different percentile choices might influence practical decision-making.

*I also encourage the authors to further emphasize the distinction between participation equality and collaboration quality. The manuscript appropriately acknowledges that equal participation does not necessarily imply productive collaboration, yet this important point could be discussed more prominently. Groups may exhibit highly balanced turn-taking while simultaneously engaging in superficial or unproductive discussions, whereas high-quality collaborative reasoning may occasionally involve temporary participation asymmetries. Consequently, the proposed benchmark values should be interpreted as indicators of structural participation patterns rather than direct measures of educational effectiveness.

*From a methodological perspective, the procedures are generally rigorous and well documented. The estimation of the local transition probabilities appears appropriate, and the sensitivity analyses provide useful evidence regarding model stability. Nevertheless, I would appreciate further discussion regarding the exclusion of students contributing fewer than ten turns during parameter estimation. While the rationale is understandable, these minimally participating students may actually represent precisely the type of participation inequality that the proposed framework ultimately seeks to identify. The potential influence of this exclusion criterion on the estimated transition probabilities should therefore be acknowledged more explicitly.

*The interpretation of the findings is generally careful and avoids unwarranted causal claims. However, some conclusions occasionally extend beyond what the evidence can directly support. The manuscript sometimes implies that groups exceeding the benchmark values are experiencing participation inequality requiring intervention. Strictly speaking, the study demonstrates only that these groups exhibit greater inequality than would be expected under the simulated baseline conditions. Whether such deviations correspond to educationally meaningful problems remains an empirical question that future research must address. Moderating this language would improve the overall rigor of the manuscript.

Finally, while the limitations section has improved, it would benefit from a more comprehensive discussion of the study's boundaries. In particular, the authors should explicitly acknowledge the cultural specificity of the sample, the restriction to triadic groups, the exclusive reliance on turn-based indicators rather than qualitative discourse features, the dependence of the simulation on estimated transition probabilities, and the absence of external validation using independent classroom datasets.

Overall, I believe this revised manuscript represents a significant improvement over the previous submission and makes an original contribution to the literature on collaborative learning and learning analytics. The methodological innovation is noteworthy, and the proposed simulation framework has considerable potential to support future research and educational practice. Nevertheless, several conceptual and interpretative issues remain unresolved, particularly regarding the generalizability and educational interpretation of the proposed benchmark values. Addressing these concerns would substantially strengthen the manuscript and increase its impact within the field.

**********

what does this mean?). If published, this will include your full peer review and any attached files.

If you choose “no”, your identity will remain anonymous but your review may still be made public.

**Do you want your identity to be public for this peer review?**  For information about this choice, including consent withdrawal, please see our Privacy Policy

Reviewer #1: **Yes:**  AYDAN IRGATOĞLU

**********

[NOTE: If reviewer comments were submitted as an attachment file, they will be attached to this email and accessible via the submission site. Please log into your account, locate the manuscript record, and check for the action link "View Attachments". If this link does not appear, there are no attachment files.]

To ensure your figures meet our technical requirements, please review our figure guidelines: https://journals.plos.org/plosone/s/figures

You may also use PLOS’s free figure tool, NAAS, to help you prepare publication quality figures: https://journals.plos.org/plosone/s/figures#loc-tools-for-figure-preparation.

NAAS will assess whether your figures meet our technical requirements by comparing each figure against our figure specifications.

Attachment Submitted filename: report.docx

# Author comment: Author response to Decision Letter 2

**DOI:** 10.1371/journal.pone.0361205.r004

24 Sep 2026

Please see the uploaded PDF document titled “Response to reviewers-2nd-round.pdf” for our point-by-point responses to the reviewer comments.

Attachment Submitted filename: Response to reviewers-2nd-round.pdf

# Peer review: Decision Letter 2

**DOI:** 10.1371/journal.pone.0361205.r005

**Authors:** Kristiawan Indriyanto

8 Oct 2026

How unequal is unequal? Turn-taking simulation for benchmarking participation and opportunity inequality in small-group collaboration

PONE-D-26-11209R2

Dear Dr. Hu,

We’re pleased to inform you that your manuscript has been judged scientifically suitable for publication and will be formally accepted for publication once it meets all outstanding technical requirements.

Within one week, you’ll receive an e-mail detailing the required amendments. When these have been addressed, you’ll receive a formal acceptance letter and your manuscript will be scheduled for publication.

An invoice will be generated when your article is formally accepted. Please note, if your institution has a publishing partnership with PLOS and your article meets the relevant criteria, all or part of your publication costs will be covered. Please make sure your user information is up-to-date by logging into Editorial Manager at Editorial Manager® and clicking the ‘Update My Information' link at the top of the page. For questions related to billing, please contact billing support.

If your institution or institutions have a press office, please notify them about your upcoming paper to help maximize its impact. If they’ll be preparing press materials, please inform our press team as soon as possible -- no later than 48 hours after receiving the formal acceptance. Your manuscript will remain under strict press embargo until 2 pm Eastern Time on the date of publication. For more information, please contact onepress@plos.org.

Kind regards,

Kristiawan Indriyanto

Academic Editor

PLOS One

Additional Editor Comments (optional):

Reviewers' comments:

Reviewer's Responses to Questions

**Comments to the Author**

Reviewer #1: All comments have been addressed

**********

2. Is the manuscript technically sound, and do the data support the conclusions?

Reviewer #1: Yes

**********

3. Has the statistical analysis been performed appropriately and rigorously?

Reviewer #1: Yes

**********

4. Have the authors made all data underlying the findings in their manuscript fully available?

The PLOS Data policy

Reviewer #1: Yes

**********

5. Is the manuscript presented in an intelligible fashion and written in standard English?

Reviewer #1: Yes

**********

Reviewer #1: (No Response)

**********

what does this mean?). If published, this will include your full peer review and any attached files.

If you choose “no”, your identity will remain anonymous but your review may still be made public.

**Do you want your identity to be public for this peer review?**  For information about this choice, including consent withdrawal, please see our Privacy Policy

Reviewer #1: **Yes:**  Aydan IRGATOĞLU

**********

# Editor report: Acceptance letter

**DOI:** 10.1371/journal.pone.0361205.r006

**Authors:** Kristiawan Indriyanto

PONE-D-26-11209R2

PLOS One

Dear Dr. Hu,

I'm pleased to inform you that your manuscript has been deemed suitable for publication in PLOS One. Congratulations! Your manuscript is now being handed over to our production team.

At this stage, our production department will prepare your paper for publication. This includes ensuring the following:

* All references, tables, and figures are properly cited

* All relevant supporting information is included in the manuscript submission,

* There are no issues that prevent the paper from being properly typeset

You will receive further instructions from the production team, including instructions on how to review your proof when it is ready. Please keep in mind that we are working through a large volume of accepted articles, so please give us a few days to review your paper and let you know the next and final steps.

Lastly, if your institution or institutions have a press office, please let them know about your upcoming paper now to help maximize its impact. If they'll be preparing press materials, please inform our press team within the next 48 hours. Your manuscript will remain under strict press embargo until 2 pm Eastern Time on the date of publication. For more information, please contact onepress@plos.org.

You will receive an invoice from PLOS for your publication fee after your manuscript has reached the completed accept phase. If you receive an email requesting payment before acceptance or for any other service, this may be a phishing scheme. Learn how to identify phishing emails and protect your accounts at https://explore.plos.org/phishing.

If we can help with anything else, please email us at customercare@plos.org.

Thank you for submitting your work to PLOS One and supporting open access.

Kind regards,

PLOS One Editorial Office Staff

on behalf of

Dr. Kristiawan Indriyanto

Academic Editor

PLOS One
